# Sex differences in islet stress responses support female β cell resilience

**DOI:** 10.1016/j.molmet.2023.101678

**Published:** 2023-01-20

**Authors:** George P. Brownrigg, Yi Han Xia, Chieh Min Jamie Chu, Su Wang, Charlotte Chao, Jiashuo Aaron Zhang, Søs Skovsø, Evgeniy Panzhinskiy, Xiaoke Hu, James D. Johnson, Elizabeth J. Rideout

**Affiliations:** Department of Cellular and Physiological Sciences, Life Sciences Institute, The University of British Columbia, 2350 Health Sciences Mall, Vancouver, BC, V6T 1Z3, Canada

**Keywords:** Pancreatic islets, β cells, Diabetes mellitus, Endoplasmic reticulum stress, Protein synthesis, Transcriptomics

## Abstract

**Objective:**

Pancreatic β cells play a key role in maintaining glucose homeostasis; dysfunction of this critical cell type causes type 2 diabetes (T2D). Emerging evidence points to sex differences in β cells, but few studies have examined male-female differences in β cell stress responses and resilience across multiple contexts, including diabetes. Here, we address the need for high-quality information on sex differences in β cell and islet gene expression and function using both human and rodent samples.

**Methods:**

In humans, we compared β cell gene expression and insulin secretion in donors with T2D to non-diabetic donors in both males and females. In mice, we generated a well-powered islet RNAseq dataset from 20-week-old male and female siblings with similar insulin sensitivity. Our unbiased gene expression analysis pointed to a sex difference in the endoplasmic reticulum (ER) stress response. Based on this analysis, we hypothesized female islets would be more resilient to ER stress than male islets. To test this, we subjected islets isolated from age-matched male and female mice to thapsigargin treatment and monitored protein synthesis, cell death, and β cell insulin production and secretion. Transcriptomic and proteomic analyses were used to characterize sex differences in islet responses to ER stress.

**Results:**

Our single-cell analysis of human β cells revealed sex-specific changes to gene expression and function in T2D, correlating with more robust insulin secretion in human islets isolated from female donors with T2D compared to male donors with T2D. In mice, RNA sequencing revealed differential enrichment of unfolded protein response pathway-associated genes, where female islets showed higher expression of genes linked with protein synthesis, folding, and processing. This differential expression was physiologically significant, as islets isolated from female mice were more resilient to ER stress induction with thapsigargin. Specifically, female islets showed a greater ability to maintain glucose-stimulated insulin production and secretion during ER stress compared with males.

**Conclusions:**

Our data demonstrate sex differences in β cell gene expression in both humans and mice, and that female β cells show a greater ability to maintain glucose-stimulated insulin secretion across multiple physiological and pathological contexts.

## Highlights

• Islet β cells from female donors living with T2D have more insulin secretion than males.
• Islets from female mice show higher expression of unfolded protein response genes.
• Islets from female mice show better recovery of protein synthesis and survival after ER stress.
• Islets from female mice maintain better insulin secretion during ER stress.
• Islets from female mice islets show distinct transcriptomic and proteomic responses to ER stress.

## 1 Introduction

Pancreatic β cells make and secrete insulin, an essential hormone required to maintain whole-body glucose homeostasis. Emerging evidence from multiple species points to biological sex as an important, but often overlooked, factor that affects β cell biology [[1], [2], [3], [4], [5], [6]]. Large-scale surveys of gene expression in mice and humans show that differences exist between the sexes in the pancreas [[7], [8], [9]], in islets [10], and in β cells specifically [4,11]. Humans also have a sex-specific β cell gene expression response to aging [12], and show male-female differences in pancreatic β cell number [6]. With respect to β cell function, most data from rodent and human studies suggests glucose-stimulated insulin secretion is higher in females than in males [5,10,[13], [14], [15], [16]]. While male-female differences in peripheral insulin sensitivity [15,[17], [18], [19], [20], [21], [22], [23], [24], [25], [26], [27]] may contribute to these differences, sex-biased insulin secretion in humans persists in the context of equivalent insulin sensitivity between males and females [5]. Whether sex differences in other aspects of β cell gene expression and function are similarly independent of insulin sensitivity in rodents and humans remains unclear, as insulin sensitivity is not routinely monitored across datasets showing sex differences in β cell biology.

Biological sex also affects the risk of developing T2D. Across many population groups, men are at a higher risk of developing T2D than women [[28], [29], [30], [31]]. Some of this differential risk is explained by lifestyle and cultural factors [[31], [32], [33]]. Biological sex also plays a role; however, as the male-biased risk of developing diabetes-like phenotypes is conserved across multiple animal models [22,[34], [35], [36], [37], [38], [39]]. Despite a dominant role for β cell function in T2D pathogenesis [40,41], T2D- and stress-associated changes to β cell gene expression and function in each sex remain largely unexplored, as most studies on these topics do not include biological sex as a variable in their analysis [[42], [43], [44], [45], [46], [47], [48], [49]]. Collecting detailed knowledge of β cell gene expression and function from each sex under physiological and pathological conditions is therefore a key first step toward understanding whether male-female differences in this important cell type may contribute to T2D risk.

The overall goal of our study was to provide detailed knowledge of β cell gene expression and function in both males and females across multiple contexts to advance our understanding of sex differences in this important cell type. Our data show male-female differences in islet and β cell gene expression and stress responses in both humans and mice. These differences contribute to sex differences in β cell resilience, where we find female β cells show a greater ability to maintain glucose-stimulated insulin secretion in response to stress and T2D in mice and humans, respectively. Given that an insulin tolerance test indicated similar insulin sensitivity between the male and female mice used in our study, our findings suggest biological sex is an important variable to consider in studies on islet and β cell function.

## 2 Materials and methods

### 2.1 Animals

Mice were bred in-house or purchased from the Jackson Laboratory. Unless otherwise stated, islets were isolated from C57BL/6J mice aged 20–24 weeks. Animals were housed and studied in the UBC Modified Barrier Facility using protocols approved by the UBC Animal Care Committee and in accordance with international guidelines. Mice were housed on a 12-hour light/dark cycle with food and drinking water *ad libitum*. Mice were fed a regular chow diet (LabDiet #5053); 24.5% energy from protein, 13.1% energy from fat, and 62.4% energy from carbohydrates.

### 2.2 Islet isolation, culture, dispersion and treatment

Mouse islet isolations were performed by ductal collagenase injection followed by filtration and hand-picking, using modifications of the protocol described by Salvalaggio [50]. Islets recovered overnight, in islet culture media (RPMI media with 11.1 mM d-glucose supplemented with 10% vol/vol fetal bovine serum (FBS) (Thermo: 12483020) and 1% vol/vol Penicillin-Streptomycin (P/S) (GIBCO: 15140-148)) at 37 °C with 5% CO_2_. After four washes with Minimal Essential Medium [l-glutamine, calcium and magnesium free] (Corning: 15-015 CV) islets were dispersed with 0.01% trypsin and resuspended in islet culture media. Cell seedings were done as per the experimental procedure (protein synthesis: 20,000 cells per well, live cell imaging: 5,000 cells per well). ER stress was induced by treating islets with the SERCA inhibitor thapsigargin. For assays less than 24 h, we used (11.1 mM d-glucose RPMI, 1% vol/vol P/S). For assays greater than 24 h we used (11.1 mM d-glucose RPMI, 1% vol/vol P/S, 10% vol/vol FBS).

### 2.3 Analysis of protein synthesis

Dispersed islets were seeded into an optical 96-well plate (Perkin Elmer) at a density of approximately 20,000 cells per well in islet culture media (11.1 mM d-glucose RPMI, 1% vol/vol P/S, 10% vol/vol FBS). 24 h after seeding, treatments were applied in fresh islet culture media (11.1 mM d-glucose RPMI, 1% vol/vol P/S). After incubation, fresh culture media was applied (11.1 mM d-glucose RPMI, 1% vol/vol P/S), then supplemented with 20 μM OPP (Invitrogen) and respective drug treatments. The assay was performed according to manufacturer's instructions. Cells were imaged at 10× with an ImageXpress^MICRO^ high-content imager and analyzed with MetaXpress (Molecular Devices) to quantify the integrated staining intensity of OPP-Alexa Fluor 594 in cells identified by NuclearMask Blue Stain.

### 2.4 Live cell imaging

Dispersed islets were seeded into 384-well plates (Perkin Elmer) at a density of approximately 5,000 cells per well. Islet viability was measured with the TC20 Automated Cell Counter (Bio-Rad: 1450102) with Trypan Blue (Bio-Rad: 1450021). Islets were allowed to adhere for 48 h in islet culture media (11.1 mM d-glucose RPMI, 1% vol/vol P/S, 10% vol/vol FBS). Cells were stained with Hoechst 33342 (Sigma–Aldrich) (0.05 μg/mL) and propidium iodide (Sigma–Aldrich) (0.5 μg/mL) for 1 h in islet culture media (11.1 mM d-glucose RPMI, 1% vol/vol P/S, 10% vol/vol FBS) prior to the addition of treatments and imaging. 384-well plates were placed into the environmentally-controlled (37 °C, 5% CO2) ImageXpress^MICRO^ high content imaging system. To measure cell death, islet cells were imaged every 2 h for 84 h. MetaXpress software was used to quantify cell death, defined as the number of Propidium Iodide-positive/Hoechst 33342-positive cells. To measure *Ins2* gene activity, dispersed islets from *Ins2*^GFP/WT^ mice aged 21–23 weeks were used [51]. Islet cells were imaged every 30 min for 60 h. MetaXpress analysis software and custom R scripts were used to perform single-cell tracking of *Ins2*^GFP/WT^ β cells as previously described [51].

### 2.5 Western blot

After overnight recovery, islets were split equally per mouse into islet culture media (11.1 mM d-glucose RPMI, 1% vol/vol P/S, 10% vol/vol FBS) resulting in ∼100–150 islets per condition. Islets were treated for 24 h with DMSO or 1 μM Tg in islet culture media then sonicated in RIPA lysis buffer (150 mM NaCl, 1% Nonidet P-40, 0.5% DOC, 0.1% SDS, 50 mM Tris (pH 7.4), 2 mM EGTA, 2 mM Na_3_VO_4_, and 2 mM NaF supplemented with complete mini protease inhibitor cocktail (Roche, Laval, QC)). Equal protein amounts (8–10 μg of protein) in equal volumes were loaded for each experiment. Protein lysates were incubated in Laemmli loading buffer (Thermo, J61337AC) at 95°C for 5 min and resolved by SDS-PAGE. Proteins were then transferred to PVDF membranes (BioRad, CA) and probed with antibodies against HSPA5 (1:1000, Cat. #3183, Cell Signalling), eIF2α (1:1000, Cat. #2103, Cell Signalling), phospho-eIF2α (1:1000, Cat. #3398, Cell Signalling), IRE1α (1:1000, Cat. #3294, Cell Signalling), phospho-IRE1α (1:1000, Cat. #PA1-16927, Thermo Fisher Scientific), CHOP (1:1000, #ab11419, Abcam), β-actin (1:1000, NB600-501, Novus Biologicals). The signals were detected by secondary HRP-conjugated antibodies (Anti-mouse, Cat. #7076; Anti-rabbit, Cat. #7074; CST) and either Pierce ECL Western Blotting Substrate (Thermo Fisher Scientific) or Forte (Immobilon). Protein band intensities were quantified using Image Studio (LI-COR).

### 2.6 Islet secretion and content

Glucose-stimulated insulin/proinsulin production and secretion were assessed using size-matched islets (five islets per well, in triplicate) seeded into 96-well V-bottom Tissue Culture Treated Microplates (Corning: #CLS3894). Islets were allowed to adhere for 48 h in culture media (11.1 mM d-glucose RPMI, 1% vol/vol P/S, 10% vol/vol FBS), based on a published method [52], as this protocol permits analysis of large sample numbers and treatments, and minimizes islet loss. Adherent islets were washed with Krebs–Ringer Buffer (KRB; 129 mM NaCl, 4.8 mM KCl, 1.2 mM MgSO_4_, 1.2 mM KH_2_PO_4_, 2.5 mM CaCl_2_, 5 mM NaHCO_3_, 10 mM HEPES, 0.5% bovine serum albumin) containing 3 mM glucose then pre-incubated for 4 h in 3 mM glucose KRB. 1 μM Tg was added to the 3 mM low glucose pre-incubation buffer 4 h prior, 2 h prior, or at the start of the low glucose incubation period. Islets were incubated in KRB with 3 mM glucose then 20 mM glucose for 45 min each. Supernatant was collected after each stimulation. Islet insulin and proinsulin content was extracted by freeze-thawing in 100 μL of acid ethanol, then the plates were shaken at 1200 rpm for 10 min at 4°C to lyse the islets. Insulin was measured by Rodent Insulin Chemiluminescent ELISA (ALPCO: 80-INSMR) and proinsulin by Rat/Mouse Proinsulin ELISA (Mercodia: 10-1232-01). Measurements were performed on a Spark plate reader (TECAN).

### 2.7 Blood collection and *in vivo* analysis of glucose homeostasis and insulin secretion

Mice were fasted for 6 h prior to glucose and insulin tolerance tests. During glucose and insulin tolerance tests, tail blood was collected for blood glucose measurements using a glucometer (One Touch Ultra 2 Glucometer, Lifescan, Canada). For intraperitoneal (i.p.) glucose tolerance tests, the glucose dose was 2 g glucose/kg of body mass. For insulin tolerance tests, the insulin dose was 0.75U insulin/kg body mass. For measurements of *in vivo* glucose-stimulated insulin secretion, femoral blood was collected after i.p. injection of 2 g glucose/kg body mass. Blood samples were kept on ice during collection, centrifuged at 2000 rpm for 10 min at 4°C and stored as plasma at −20 °C. Plasma samples were analysed for insulin using Rodent Insulin Chemiluminescent ELISA (ALPCO: 80-INSMR). Glucose-stimulated insulin secretion from an independent large cohort of *Insr*^f/f^:*Ins1*Cre^−/−^:nTnG^+/−^ mice reared in our facility was replotted from a published Johnson lab study [53]. Blood glucose was monitored using control and insulin-reduced mice. Control mice were *Ins1*^−/−^:*Ins2*^f/f^:mTmG + tamoxifen and *Ins1*^−/−^:*Ins2*^f/f^:*Pdx1*Cre^ERT^:mTmG + corn oil [54]. Because we confirmed there were no significant differences in blood glucose between control genotypes, the data were combined in our analysis. Insulin-reduced mice were generated by injecting *Ins1*^−/−^:*Ins2*^f/f^:Pdx1Cre^ERT^:mTmG mice with tamoxifen (3 mg/40 g body weight, dissolved in corn oil, for 4 consecutive days) at 6–8 weeks [54].

### 2.8 RNA sequencing

To assess basal transcriptional differences, islets from male and female mice (n = 9M, 8F) were snap-frozen and stored at −80°C until RNA extraction. To assess Tg-induced transcriptional changes, islets from each mouse were treated with DMSO or 1 μM Tg for 6- or 12-hours in culture media (11.1 mM d-glucose RPMI, 1% vol/vol P/S). Four groups per sex (eight groups total) were analyzed: 6 h DMSO, 6 h Tg, 12 h DMSO and 12 h Tg, n = 3–4 per group, each *n* represents pooled islet RNA from two mice. Islets were frozen at −80°C in 100 μL of RLT buffer (Qiagen) with beta mercaptoethanol (1%). RNA was isolated using RNeasy Mini Kit (Qiagen #74106) according to manufacturer's instructions. RNA from 43 to 62 islets was pooled from two mice and 19–150 ng of RNA was sequenced per pooled sample. RNA sequencing was performed at the UBC Biomedical Research Centre Sequencing Core. Sample quality control was performed using the Agilent 2100 Bioanalyzer System (RNA Pico LabChip Kit). Qualifying samples were prepped following the standard protocol for the NEBNext Ultra II Stranded mRNA (New England Biolabs). Sequencing was performed on the Illumina NextSeq 500 with Paired End 42bp × 42bp reads. Demultiplexed read sequences were then aligned to the reference sequence (UCSC mm10) using STAR aligner (v 2.5.0b) [55]. Gene differential expression was analyzed using DESeq2 R package [56]. Pathway enrichment analysis were performed using Reactome [57]. Over-representation analysis was performed using NetworkAnalyst3.0 (www.networkanalyst.ca) [58].

### 2.9 Proteomics

Islets were treated with DMSO or 1 μM Tg for 6 h in islet culture media (11.1 mM d-glucose RPMI, 1% vol/vol P/S). Two groups per sex (four groups total): 6 h DMSO and 6 h Tg, n = 5–7 per group, each *n* represents 200–240 islets pooled from two mice. Islet pellets were frozen at −80°C in 100 μL of SDS lysis buffer (4% SDS, 100 mM Tris, pH 8) and the proteins in each sample were precipitated using acetone. The University of Victoria proteomics service performed non-targeted quantitative proteomic analysis using data-independent acquisition (DIA) with LC-MS/MS on an Orbitrap mass spectrometer using 1 μg of protein. A mouse FASTA database was downloaded from Uniprot (http://uniprot.org). This file was used with the 6 gas phase fraction files from the analysis of the chromatogram library sample to create a mouse islet-specific chromatogram library using the EncyclopeDIA (v 1.2.2) software package (Searle et al., 2018). This chromatogram library file was then used to perform identification and quantitation of the proteins in the samples again using EncyclopeDIA with Overlapping DIA as the acquisition type, trypsin used as the enzyme, CID/HCD as the fragmentation, 10 ppm mass tolerances for the precursor, fragment, and library mass tolerances. The Percolator version used was 3.10. The precursor FDR rate was set to 1%. Protein abundances were log2 transformed, imputation was performed for missing values, then proteins were normalized to median sample intensities. Differential expression was analyzed using limma in Perseus [59].

### 2.10 Analysis of the transcriptome and partial proteome

Tg-induced changes to gene expression and protein levels were compared 6 h post-treatment. Log2-transformed fold change values were used to assess the congruence between our proteomics data and RNAseq data. Genes and proteins that were concordantly altered by Tg treatment at both the mRNA and protein level were searched in PubMed for relevant literature on their role in β cells. The search term used was ((“beta cell”) OR (islet) OR (“β cell”)) AND (Gene_Name). Additional annotations for all mouse proteins were downloaded from Uniprot [Dec 2022].

### 2.11 Data from HPAP

To compare sex differences in dynamic insulin secretion, data acquired was from the Human Pancreas Analysis Program (HPAP-RRID:SCR_016202) Database (https://hpap.pmacs.upenn.edu), a Human Islet Research Network (RRID:SCR_014393) consortium (UC4-DK-112217, U01-DK-123594, UC4-DK-112232, and U01-DK-123716).

### 2.12 Statistical analysis

Statistical analyses and data presentation were carried out using GraphPad Prism 9 (GraphPad Software, San Diego, CA, USA) or R (v 4.1.1). Correlation plots were generated using the corrplot R package (v 0.92) with default settings [60]. All R codes are published on github (https://github.com/johnsonlabubc/ER-Stress-in-Mouse-Beta-Cells-Data-Analysis). Student's *t*-tests or two-way ANOVAs were used for parametric data. A Mann–Whitney test was used for non-parametric data. Statistical tests are indicated in the figure legends. For all statistical analyses, differences were considered significant if the *p*-value was less than 0.05. ∗: p < 0.05; ∗∗p < 0.01; ∗∗∗p < 0.001. Data were presented as means ± SEM with individual data points from biological replicates.

## 3 Results

### 3.1 Sex differences in β cell transcriptional and functional responses in ND and T2D human islets

**Figure 1:**
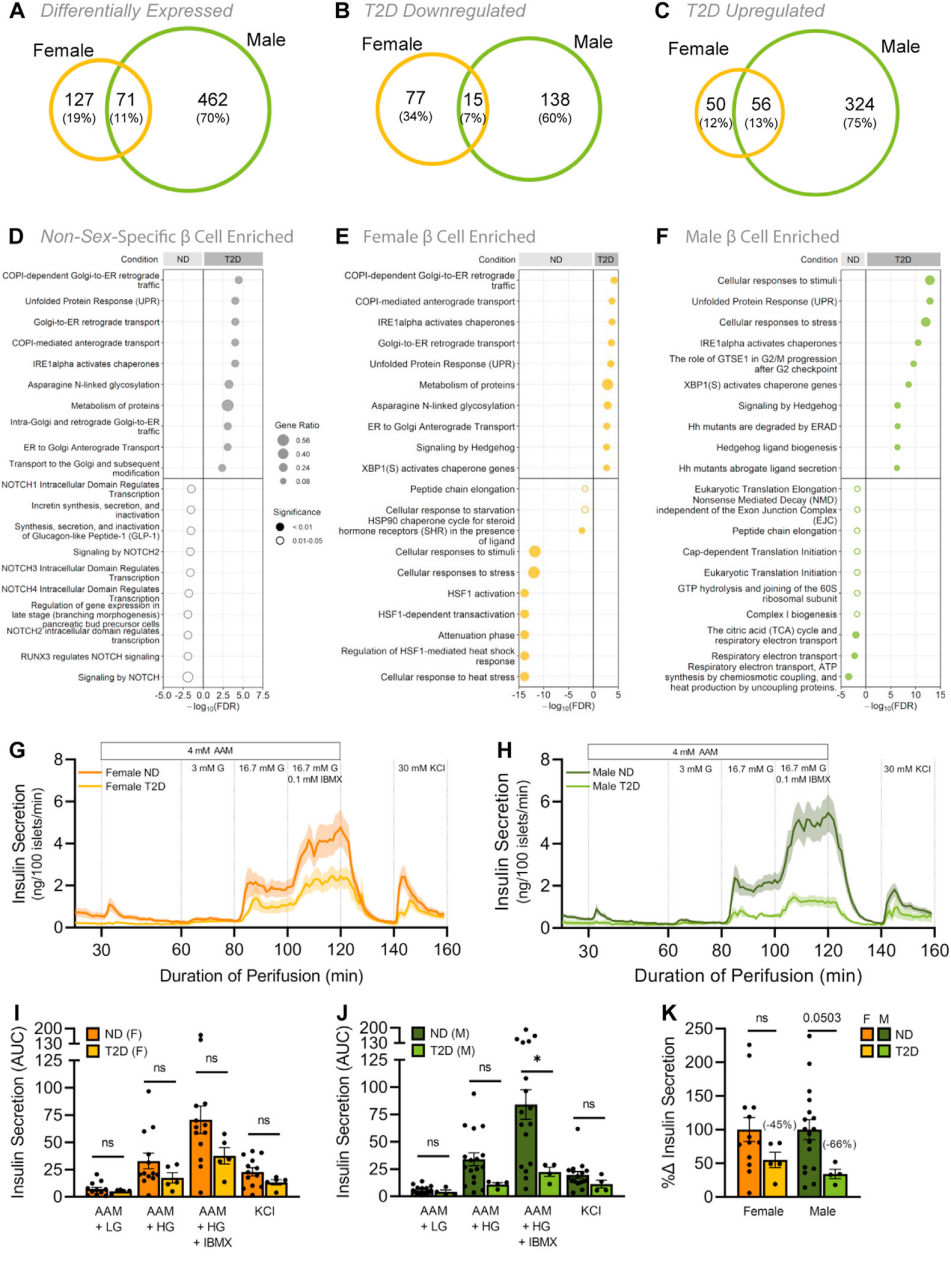
**Sex differences in human islet transcriptomic and functional responses in type 2 diabetes.** scRNAseq data from male and female human β cells. For donor metadata see Supplementary file 8. (A–C) Venn diagrams compare the number of significantly differentially expressed genes between ND and T2D donors (*p*-adj<0.05). All differentially expressed genes (A), downregulated genes (B), upregulated genes (C) in T2D human β cells. For complete gene lists see Supplementary file 1 and 2. (D–F) Top 10 significantly enriched Reactome pathways (ND vs T2D) from non-sex-specific (D), female (E), or male (F) significantly differentially expressed genes (*p*-adj< 0.05). Gene ratio is calculated as *k/n*, where *k* is the number of genes identified in each Reactome pathway, and *n* is the number of genes from the submitted gene list participating in any Reactome pathway. For complete Reactome pathway lists see Supplementary file 2. (G–K) Human islet perifusion data from the Human Pancreas Analysis Program in ND and T2D donor islets in females (F, I) and males (G, H). 3 mM glucose (3 mM G); 16.7 mM glucose (16.7 mM G); 0.1 mM isobutylmethylxanthine (0.1 mM IBMX); 30 mM potassium chloride (30 mM KCl); 4 mM amino acid mixture (4 mM AAM; mM: 0.44 alanine, 0.19 arginine, 0.038 aspartate, 0.094 citrulline, 0.12 glutamate, 0.30 glycine, 0.077 histidine, 0.094 isoleucine, 0.16 leucine, 0.37 lysine, 0.05 methionine, 0.70 ornithine, 0.08 phenylalanine, 0.35 proline, 0.57 serine, 0.27 threonine, 0.073 tryptophan, and 0.20 valine, 2 mM glutamine). (I–K) Quantification of area under the curve (AUC) is shown for the various stimulatory media in females (I), males (J) and donors with T2D (K). (I) In females, insulin secretion from ND islets was not significantly higher than T2D islets under any culture condition (p = 0.4806 [AAM + LG], p = 0.2270 [AAM + HG], p = 0.1384 [AAM + HG + IBMX], and p = 0.1465 [KCl]; unpaired Student's *t*-test). (J) In males, insulin secretion from ND islets was significantly higher than T2D islets under 4 mM AAM +16.7 mM glucose (HG) + 0.1 mM IBMX stimulation (p = 0.0442 [AAM + HG + IBMX]; unpaired Student's *t*-test), but not in other conditions (p = 0.5315 [AAM + LG], p = 0.0818 [AAM + HG], and p = 0.2259 [KCl]; unpaired Student's *t*-test). (K) Total insulin secretion showed a trend toward lower secretion in T2D male islets than ND male islets (p = 0.1514 and p = 0.0503 for females and males, respectively; unpaired Student's *t*-test). ∗ indicates p < 0.05; ns indicates not significant; error bars indicate SEM.

Gene expression studies on human pancreas and islets identify significant sex differences in gene expression [7,10]. Indeed, >1500 genes expressed in the pancreas show sex-biased expression [7]. While our dataset contained fewer individuals, we also note sex differences in mRNA levels of many genes in β cells (Supplementary file 1). Given this differential gene expression, we wanted to define β cell-specific gene expression changes in T2D in each sex. We therefore used a recently-compiled meta-analysis of publicly available scRNAseq datasets from male and female human islets [61]. Our goal was to use sex-based analysis to determine whether β cell gene expression changes in T2D are shared between the sexes. In line with prior reports [12], β cells from non-diabetic (ND) and T2D donors showed significant transcriptional differences. In β cells isolated from female T2D donors, mRNA levels of 127 genes were significantly different from ND female donors (77 downregulated, 50 upregulated in T2D) (Figure 1A–C). In β cells isolated from male T2D donors, 462 genes were differentially expressed compared with male ND donors (138 downregulated, 324 upregulated in T2D) (Figure 1A–C). Of the 660 genes that were differentially regulated in T2D, 71 were differentially regulated in both males and females (15 downregulated, 56 upregulated in T2D) (Figs. S1A–C); however, the fold change for these 71 shared genes was different between males and females (Fig. S1A; Supplementary file 2). This suggests that for shared genes, the magnitude of gene expression changes in T2D was not the same between the sexes. Beyond shared genes, we observed that the majority of differentially expressed genes in T2D (589/660) were unique to either males or females (Figs. S1B,C; Supplementary file 2). Indeed, the most prominent gene expression changes in T2D were found in genes that were unique to one sex (Figs. S2A,B; Supplementary file 2). While these data do not address the reasons for the sex-biased risk of T2D, and could reflect differences in medication [[62], [63], [64], [65]], age [12,16,[66], [67], [68]], and body mass index [15,68], our data suggest biological sex influences β cell gene expression in T2D.

To determine which biological pathways were altered in β cells of T2D donors from each sex, we performed pathway enrichment analysis. Genes that were upregulated in β cells isolated from T2D donors included genes involved in Golgi-ER transport and the unfolded protein response (UPR) pathways (Figure 1D–F; Supplementary file 2). While these biological pathways were significantly upregulated in T2D in both males and females, ∼75% of the differentially regulated genes in these categories were unique to each sex (Table 1). Genes that were downregulated in β cells from T2D donors revealed further differences between the sexes: biological pathways downregulated in β cells from female T2D donors included cellular responses to stress and to stimuli (Figure 1E; Supplementary file 2), whereas β cells from male T2D donors showed downregulation of pathways associated with respiratory electron transport and translation initiation (Figure 1F; Supplementary file 2). Our analysis therefore suggests that sex-biased β cell gene expression responses to T2D may influence different cellular processes in males and females.

**Table 1:** **Human β cell pathway gene numbers.** The number of genes corresponding to each T2D upregulated pathway in males, females or both sexes.

Pathway Name	Number of Pathway Genes		
	Unique Male	Common	Unique Female
Asparagine N-linked glycosylation	16	9	4
Cellular responses to stimuli	49	9	6
Cellular responses to stress	47	9	6
COPI-dependent Golgi-to-ER retrograde traffic	7	6	2
COPI-mediated anterograde transport	7	5	2
ER to Golgi Anterograde Transport	10	5	2
Golgi-to-ER retrograde transport	7	6	2
Hedgehog ligand biogenesis	12	4	1
Hh mutants abrogate ligand secretion	12	3	1
Hh mutants are degraded by ERAD	12	3	1
IRE1alpha activates chaperones	9	3	1
Metabolism of proteins	69	20	13
Signaling by Hedgehog	16	6	2
The role of GTSE1 in G2/M progression after G2 checkpoint	14	4	1
Unfolded Protein Response (UPR)	13	3	1
XBP1(S) activates chaperone genes	8	3	1

The sex-biased β cell transcriptional response in T2D prompted us to compare glucose-stimulated insulin secretion in each sex from ND and T2D human islets using data from the Human Pancreas Analysis Program database [69]. In ND donors, islets from males and females showed similar patterns of insulin secretion in response to various stimulatory media (Figure 1G,H). In donors with T2D, we found that insulin secretion was impaired to a greater degree in islets from males than in females (Figure 1G–K). This difference cannot be fully attributed to a sex difference in disease severity, as our analysis of donor characteristics revealed no significant correlation between sex and HbA1c (Fig. S3A). Indeed, in male but not female islets, insulin secretion was lower in donors with T2D following stimulation with both high glucose and IBMX (Figure 1I,J), which potentiates insulin secretion by increasing cAMP levels to a similar degree as the incretins [70]. Human islets from female donors with T2D therefore show a greater ability to maintain glucose-stimulated insulin secretion than islets from males with T2D (Figure 1K). Indeed, while diabetes status was the main donor characteristic that correlated with changes in insulin secretion (Fig. S3B), we noted that in T2D sex and age were two donor characteristics showing trends toward an effect on insulin secretion (Fig. S3A). Combined with our β cell gene expression data, these findings suggest that β cell transcriptional and functional responses in T2D are not shared between the sexes.

### 3.2 Sex differences in UPR-associated gene expression in mouse islets

Our unbiased analysis of human β cell gene expression and function in T2D revealed differences between male and female donors with T2D. Because human β cell gene expression and function can be affected by factors such as peripheral insulin sensitivity, disease processes, and medication [31,33], we investigated sex differences in β cell gene expression and function in another context. We generated a well-powered islet RNAseq dataset from 20-week-old male and female C57BL/6J mice. We used an insulin tolerance test (ITT) to show that insulin sensitivity was similar between the sexes at this age (Fig. S4A); however, we acknowledge that the ITT may not be as sensitive as a hyperinsulinemic-euglycemic clamp in detecting modest sex differences in insulin sensitivity.

**Figure 2:**
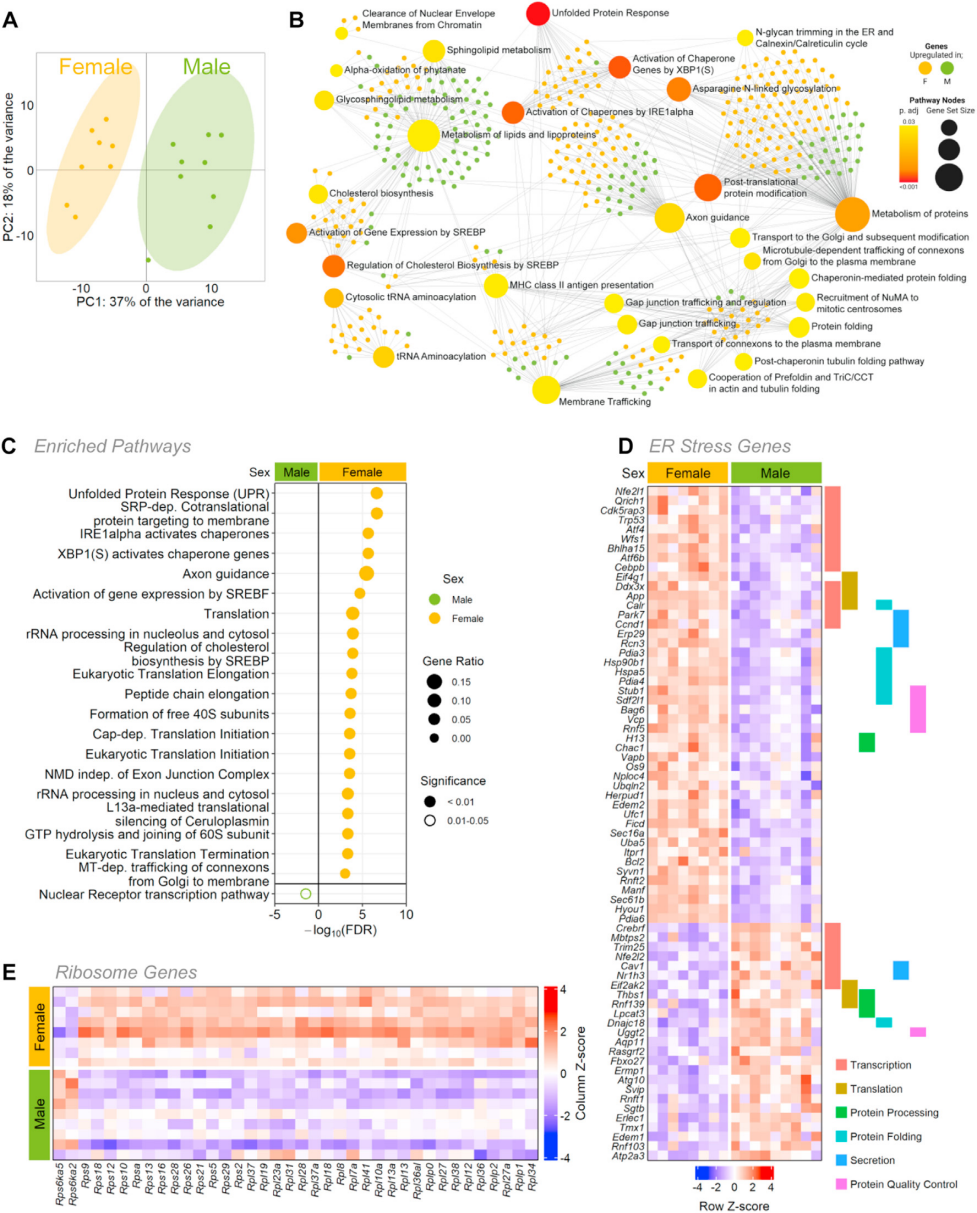
**Sex-biased gene expression in mouse islet bulk RNAseq**. (A) Principal component analysis (PCA) of RNAseq data from male and female mouse islets. (B) Over-representation analysis (ORA) of all significantly differentially expressed genes (*p*-adj <0.01) from male and female mouse islets. Top 30 enriched KEGG pathways (large nodes; size = proportional to connections, darker red color = greater significance) and associated genes (small nodes; green = male enriched, yellow = female enriched). (C) Top significantly enriched Reactome pathways from the top 1000 significantly differentially expressed genes. (*p*-adj <0.01) for males and females. Gene ratio is calculated as k/n, where k is the number of genes identified in each Reactome pathway, and n is the number of genes from the submitted gene list participating in any Reactome pathway. For complete Reactome pathway lists see Supplementary file 3. (D) All transcripts of differentially expressed genes under the gene ontology term “Cellular response to ER stress” (GO:0034976) and genes labeled by their role in transcription, translation, protein processing, protein folding, secretion and protein quality control. (E) All transcripts of differentially expressed ribosomal genes.

Principal component analysis and unsupervised clustering clearly separated male and female islets on the basis of gene expression (Figure 2A; Fig. S5A). We found that 17.7% (3268/18938) of genes were differentially expressed between the sexes (1648 upregulated in females, 1620 upregulated in males), in line with estimates of sex-biased gene expression in other tissues [71,72]. Overrepresentation and pathway enrichment analysis both identified UPR-associated pathways as a biological process that differed significantly between the sexes, where the majority of genes in this category were enriched in female islets (Figure 2B,C; Supplementary file 3). Additional genes that were enriched in female islets were those associated with the gene ontology term “Cellular response to endoplasmic reticulum stress” (GO:0034976), which included many genes involved in regulating protein synthesis (Figure 2D). For example, females showed significantly higher levels of most ribosomal protein genes (Figure 2E). Further genes enriched in females included those associated with protein folding, protein processing, and quality control (Figure 2D). Given that protein synthesis, processing, and folding capacity are intrinsically important for multiple islet cell types [[73], [74], [75], [76]], including β cells [77,78], this suggests female islets may have a larger protein production and folding capacity than male islets.

### 3.3 Female islets are more resilient to endoplasmic reticulum stress in mice

**Figure 3:**
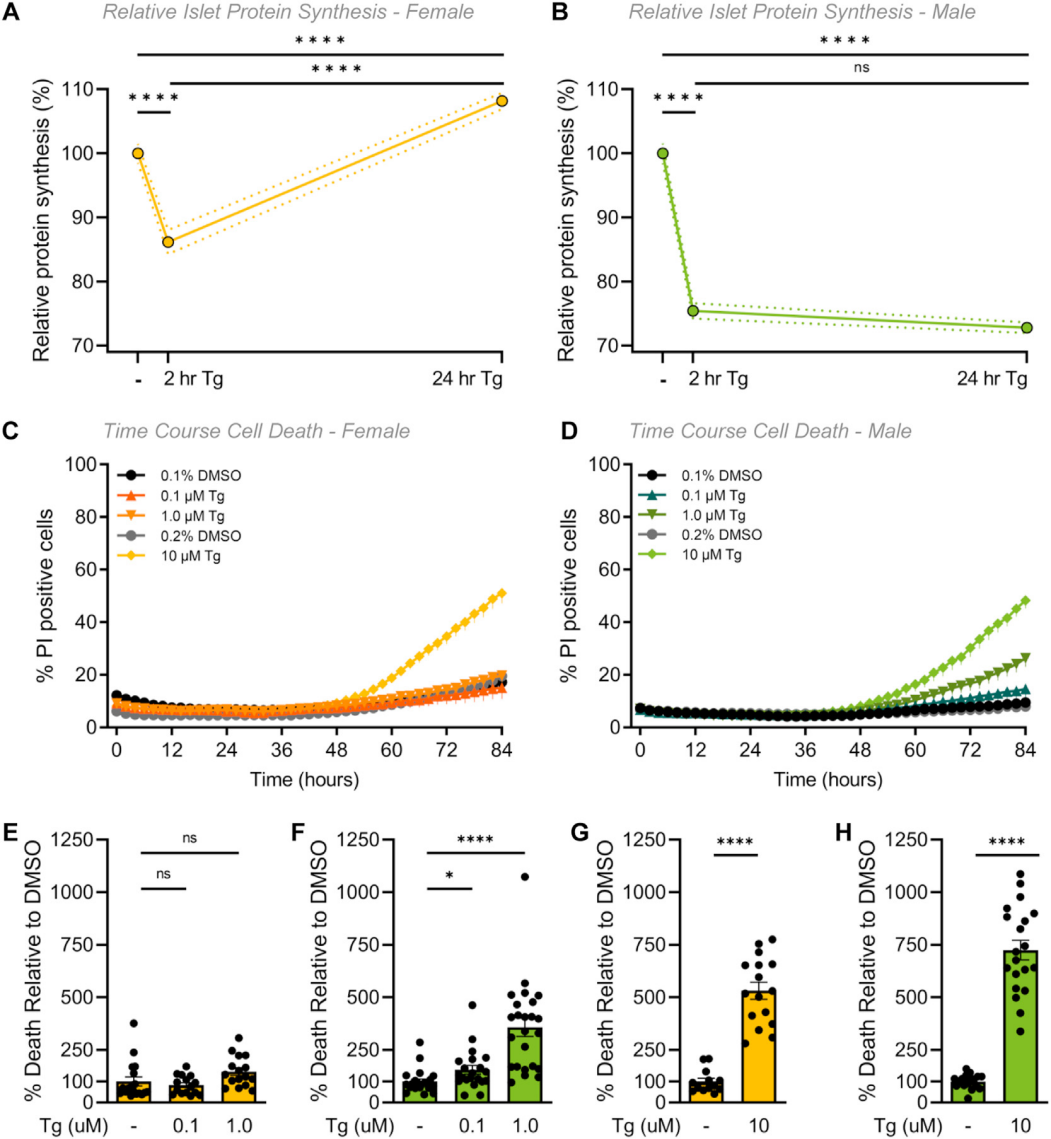
**Sex differences in mouse islet ER stress-associated phenotypes.** (A, B) Protein synthesis was quantified in dispersed islet cells from 20-week-old male and female B6 mice after treatment with 1 μM Tg for 2- or 24-hours. (A) In female islet cells, protein synthesis was significantly lower after a 2-hour Tg treatment compared to control (p < 0.0001; one-way ANOVA followed by Tukey HSD test), significantly higher after a 24-hour Tg treatment compared to a 2-hour Tg treatment (p < 0.0001; one-way ANOVA followed by Tukey HSD test) and recovered to a significantly higher level than control levels p < 0.0001; one-way ANOVA followed by Tukey HSD test). (B) In male islet cells, protein synthesis was significantly lower after a 2- and 24-hour Tg treatment compared to control (p < 0.0001 for both treatments; one-way ANOVA followed by Tukey HSD test) and was not significantly different after a 24-hour treatment compared to a 2-hour Tg treatment p = 0.3022; one-way ANOVA followed by Tukey HSD test). The magnitude of protein synthesis repression and recovery was significantly different in all sex:treatment interactions (p = 0.0015 [DMSO-2hr], p < 0.0001 [DMSO-24hr], p < 0.0001 [2hr–24hr]; two-way ANOVA followed by Tukey HSD test). (C–H) Quantification of propidium iodide (PI) cell death assay of dispersed islets from 20-week-old male and female B6 mice treated with thapsigargin (0.1 μM, 1 μM or 10 μM Tg) or DMSO for 84 h n = 4–6 mice, >1000 cells per group. Percentage (%) of PI positive cells was quantified as the number of PI-positive/Hoechst 33342-positive cells in female (C) and male (D) islet cells. Relative cell death at 84 h in Tg treatments compared with DMSO treatment in females (E, G) and males (F, H). The control for both 0.1 and 1.0 μM Tg treatments is 0.1% DMSO (E, F). The control for 10 μM Tg treatment is 0.2% DMSO (G, H). In female islet cells, cell death was significantly higher in 10 μM Tg compared to control (p < 0.0001; unpaired Student's *t-*test). In male islet cells, cell death was significantly higher in 0.1, 1.0 and 10 μM Tg compared to control (p = 0.0230 [0.1 μM], p < 0.0001 [1 μM] and p < 0.0001 [10 μM]; unpaired Student's *t*-test) (D). For E-H, at 84 h the % of PI positive cells for each treatment was normalized to the DMSO control avg for each sex. ∗ indicates p < 0.05, ∗∗ indicates p < 0.01, ∗∗∗∗ indicates p < 0.0001; ns indicates not significant; error bars indicate SEM.

The burden of insulin production causes endoplasmic reticulum (ER) stress in β cells [[79], [80], [81]]. ER stress is associated with an attenuation of mRNA translation [82], and, if ER stress is prolonged, can lead to cell death [[83], [84], [85]]. Given that female islets exhibited higher expression of genes associated with protein synthesis, processing, and folding than males, and higher expression of genes associated with the UPR, which is activated in response to ER stress [86], we examined global protein synthesis rates in male and female islets under basal conditions and during ER stress. We incubated islets with O-propargyl-puromycin (OPP), which is incorporated into newly-translated proteins and can be ligated to a fluorophore. Using this technique, we monitored the accumulation of newly-synthesized islet proteins with single-cell resolution (Fig. S6A). In basal culture conditions, male islet cells had significantly greater protein synthesis rates compared with female islet cells (Fig. S6B). To investigate islet protein synthesis under ER stress in each sex, we treated islets with thapsigargin (Tg), a specific inhibitor of the sarcoplasmic/endoplasmic reticulum Ca^2+^-ATPase (SERCA) that induces ER stress and the UPR by lowering ER calcium levels [83,87]. At 2 h post-Tg treatment, protein synthesis was repressed as expected in both male and female islet cells (Figure 3A,B; Fig. S6C). At 24 h post-Tg treatment, we found that protein synthesis was restored to higher-than basal levels in female islet cells, but not in male islet cells (Figure 3A,B; Fig. S6C). Importantly, a two-way ANOVA showed that recovery from protein synthesis repression was significantly different between males and females (sex:treatment interaction p < 0.0001). This suggests that while protein synthesis repression associated with ER stress was transient in female islets, this phenotype persisted for longer in male islets. Because insulin biosynthesis accounts for approximately half the total protein production in β cells [88], one potential explanation for the sex-specific recovery from protein synthesis repression is a sex difference in transcriptional changes to insulin. To test this, we quantified GFP levels in β cells isolated from male and female mice with GFP knocked into the endogenous mouse *Ins2* locus (*Ins2*^GFP/WT^) [51,89]. While ER stress induced a significant reduction in *Ins2* gene activity, this response was equivalent between the sexes. This suggests *Ins2* transcriptional changes cannot fully explain the sex difference in recovery from protein synthesis repression during ER stress (Figs. S7A–C).

Given the prolonged protein synthesis repression in males following ER stress, we next quantified cell death, another ER stress-associated phenotype [86], in male and female islets. Using a kinetic cell death assay, we observed clear sex differences in Tg-induced cell death at 0.1 μM and 1.0 μM Tg doses throughout the time course of the experiment (Figure 3C,D). Notably, viability prior to the assay was not different between males and females (Fig. S8). After 84 h of Tg treatment, no significant increase in female islet cell apoptosis was observed with either 0.1 μM or 1.0 μM Tg treatment compared with controls (Figure 3E). In contrast, cell death was significantly higher at both the 0.1 μM and the 1.0 μM doses of Tg in male islet cells compared with vehicle-only controls (Figure 3F). Our analysis shows the magnitude of Tg-induced cell death was larger in male islet cells compared with female islet cells (sex:treatment interaction p = 0.0399 [0.1 μM], p = 0.0007 [1.0 μM]). While one possible explanation for these data is that female islets are resistant to Tg-induced cell death, we found a significant increase in apoptosis in both female and male islet cells treated with 10 μM Tg (Figure 3G,H, sex:treatment interaction p = 0.0996 [0.1 μM]; data graphed separately due to different DMSO control). This suggests female islets were more resilient to mild ER stress caused by low-dose Tg than male islets.

To determine whether this increased ER stress resilience was caused by differential UPR signaling, we monitored levels of several protein markers of UPR activation including binding immunoglobulin protein (BiP), phosphorylated inositol-requiring enzyme 1 (pIRE1), phosphorylated eukaryotic initiation factor alpha (peIF2α), and C/EBP homologous protein (CHOP) [90,91] after treating male and female islets with 1 μM Tg for 24 h. We found no sex difference in UPR protein markers between male and female islets without Tg treatment (Figs. S9A–D) and observed a significant increase in levels of pIRE1α and CHOP in islets from both sexes and BiP in female islets after a 24-hour Tg treatment (Figs. S9A–D). Lack of a sex difference in protein markers suggests UPR activation by Tg treatment was similar between male and female islets at 20 weeks of age. This finding differs from the male-biased UPR activation reported in the KINGS mouse model of endogenous ER stress [37]. While one potential explanation for this discrepancy is that Tg treatment induces acute ER stress in contrast to the chronic ER stress in KINGS mice, further experiments will be needed to confirm this possibility. Of note, we reproduced the male-biased induction of BiP in islets isolated from 60-week-old male and female mice (Figs. S9E–G), suggesting that age contributes to the sex difference in UPR activation. Together, our data indicate that despite equivalent UPR activation in male and female islets treated with Tg, significant sex differences exist in ER stress-associated protein synthesis repression and cell death.

### 3.4 Female islets retain greater β cell function during ER stress in mice

**Figure 4:**
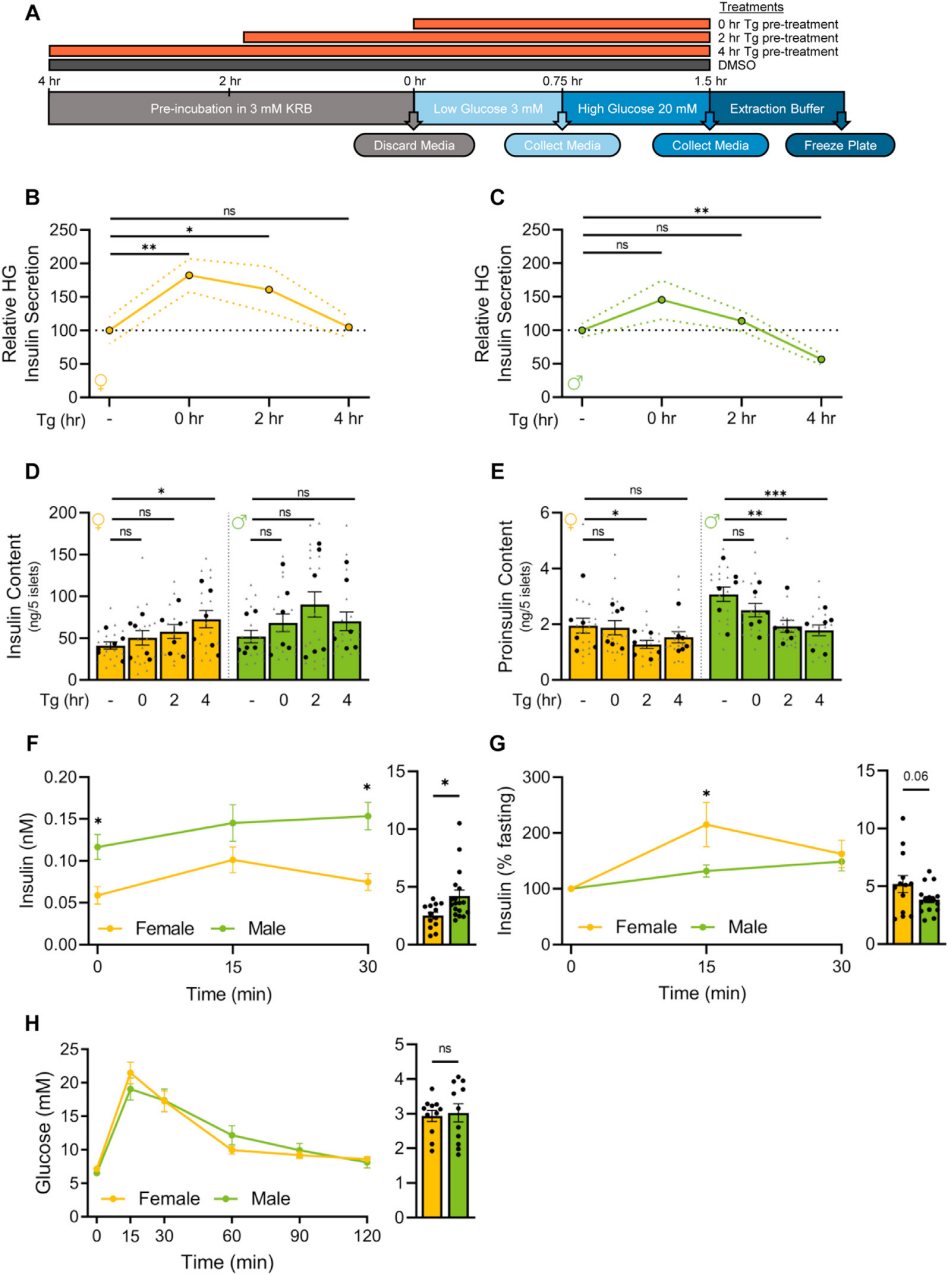
**Sex differences in *ex vivo* and *in vivo* insulin secretion**. (A) Experimental workflow of static glucose-stimulated insulin secretion. (B, C) Relative high glucose (20 mM; high glucose, HG) in treatments compared with DMSO in female (B) and male (C) islets. Female islet HG secretion was significantly higher compared with control after 0- and 2-hour Tg pre-treatments (p = 0.0083 [0-hour] and p = 0.0371 [2-hour]; Mann Whitney test). Male islet HG secretion was significantly lower compared with control after a 4-hour Tg pre-treatment (p = 0.0013; Mann Whitney test). (D) Insulin content. Female islet insulin content was significantly higher compared with control after a 4-hour Tg pre-treatment (p = 0.0269; Mann Whitney test). (E) Proinsulin content. Female islet proinsulin content was significantly lower compared with control after a 2-hour Tg pre-treatment (p = 0.0437; Mann Whitney test). Male islet proinsulin content was significantly lower compared with control after 2- and 4-hour Tg pre-treatments (p = 0.0014 [2-hour] and p = 0.0005 [4-hour]; Mann Whitney test). (F–H) Physiology measurements after a 6-hour fast in 20-week-old male and female B6 mice. (F, G) Insulin levels from glucose-stimulated insulin secretion tests (F: nM, G: % basal insulin) following a single glucose injection (2 g glucose/kg body weight, i.p). Area under the curve (AUC) calculations (n = 13 females, n = 18 males). (F) Insulin levels were significantly higher in male mice at 0 min and 30 min post injection (p = 0.0063 [0 min] and p = 0.0009 [30 min]; Student's *t*-test). AUC was significantly higher in males (p = 0.0159; Student's *t*-test). (G) Insulin levels (% baseline). Glucose-stimulated insulin secretion was significantly higher in female mice 15 min post injection (p = 0.0279; Student's *t*-test). (H) Glucose levels from glucose tolerance tests following a single glucose injection (2 g glucose/kg body weight). AUC calculations (n = 11 females, n = 11 males). For B-E, grey triangles indicate the concentration of insulin or proinsulin from five islets, black circles indicate the average values per mouse. ∗ indicates p < 0.05, ∗∗ indicates p < 0.01, ∗∗∗ indicates p < 0.001; ns indicates not significant; error bars indicate SEM.

We next examined glucose-stimulated insulin secretion in islets cultured under basal conditions and during Tg treatment (Figure 4A). In all conditions tested, high glucose significantly stimulated insulin secretion in both sexes (Fig. S10A); however, we identified sex differences in how well islets sustained glucose-stimulated insulin secretion during longer Tg treatments (Figure 4B,C, Fig. S10A,B). Female islets, in both low and high glucose, maintained robust insulin secretion during Tg treatment (Figure 4B). Specifically, we observed a significant increase in insulin secretion after short Tg treatment (0 and 2 h post-Tg), with a return to basal secretion levels 4 h post-Tg (Figure 4B). Because Tg is a drug that depletes ER calcium stores, it may induce an acute rise in cytosolic calcium that could explain this acute increase in high glucose-stimulated insulin secretion in Tg-treated samples compared with vehicle [83]. In contrast, male islets showed no significant increase in insulin secretion after short Tg treatment, and there was a significant drop in insulin secretion at 4 h post-Tg treatment (Figure 4C). This suggests female islets sustained insulin secretion for a longer period than male islets during ER stress. Given that insulin content measurements showed insulin content significantly increased during the 4-hour Tg treatment in female islets, but not male islets (Figure 4D, Fig. S10C), our data suggest one reason female islets maintain insulin secretion during ER stress is by augmenting islet insulin content. Proinsulin secretion followed similar trends to those we observed with insulin secretion (Figs. S10D,E), but Tg treatment reduced proinsulin content to a greater degree in male islets (Figure 4E). There was no sex difference in the ratio of proinsulin:insulin content at any timepoint (Fig. S10G). This suggests that in addition to a greater ability to maintain glucose-stimulated insulin secretion during ER stress, female islets also show a larger increase in insulin content and a smaller decrease in proinsulin content in this context.

To determine whether female islets have improved β cell function under ER stress in other contexts, we next monitored glucose-stimulated insulin secretion and glucose tolerance in mice at 20 weeks, an age where we demonstrated that insulin sensitivity was similar between the sexes (Figure 4F–H; Fig. S4). We found that fasting plasma insulin levels were higher in males (Figure 4F) and that the sexes showed similar glucose tolerance (Figure 4H). To determine the magnitude of the acute glucose-stimulated insulin secretion response in each sex, we normalized glucose-stimulated insulin secretion to basal insulin secretion levels. We found that females showed a trend toward higher acute glucose-stimulated insulin secretion response in C57BL/6J mice (Figure 4G), and significantly higher glucose-stimulated insulin secretion in 10- and 22-week-old *Insr*^f/f^:*Ins1*Cre^−/−^:nTnG^+/−^ mice (Figs. S11A–D; data replotted from a prior Johnson lab study [53]). These findings align with data showing higher glucose-stimulated insulin secretion from prior studies in humans and rodents [5,10,16,92,93]. We also found that fasting blood glucose levels in female mice were more resilient to the near-total insulin gene knockout in *Ins1*^−/−^*;Ins2*^fl/fl^;*Pdx1*CreER mice given tamoxifen (Fig. S11E; replotted from published [54] and unpublished Johnson lab data). We cannot rule out all potential factors that may contribute to the sex differences in blood glucose levels following near-total loss of insulin gene function (*e.g*. RNA stability, translation efficiency); however, given that the burden of insulin production [54] leads to ER stress even in normal physiological conditions, our data add further support to a model in which β cells in female mice show a greater ability to maintain glucose-stimulated insulin production and secretion during ER stress.

### 3.5 Sex differences in islet transcriptional and proteomic responses to ER stress in mice

**Figure 5:**
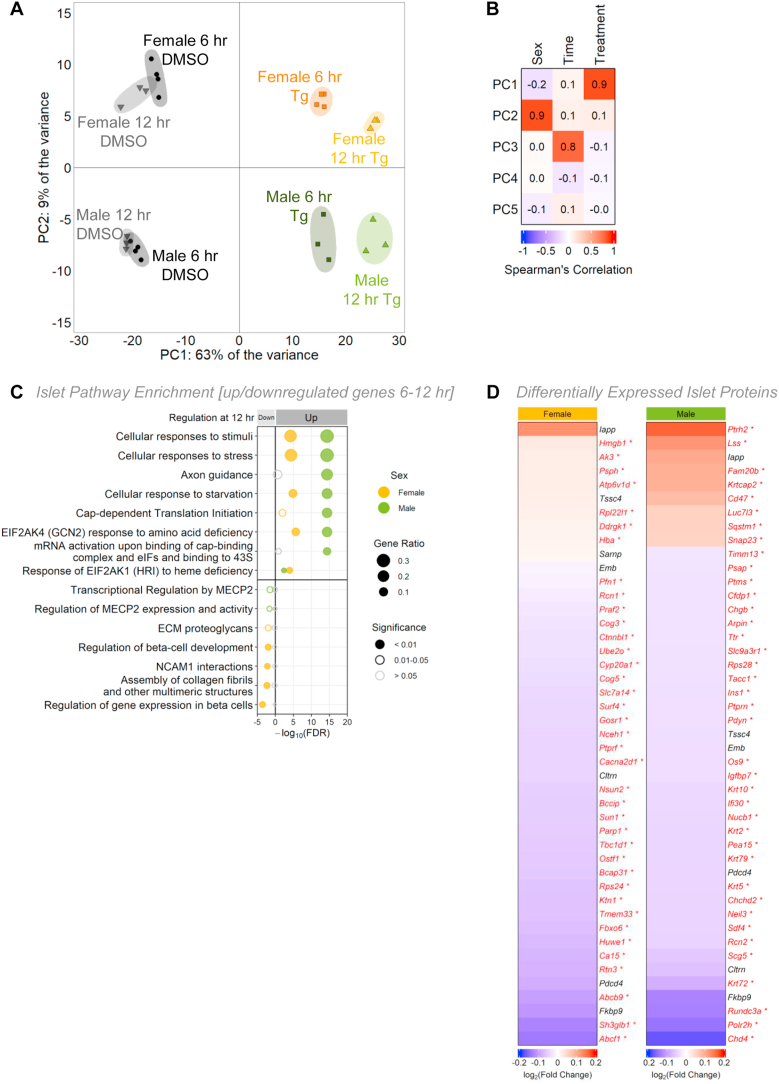
**Sex-specific transcriptomic and proteomic profiles following ER stress in mouse islets.** (A) Principal component analysis (PCA) of RNAseq data from male and female mouse islets treated with DMSO or 1 μM Tg for 6- or 12-hours. (B) Spearman correlation depicting the variance for the first 5 principal components. (C) Top significantly enriched Reactome pathways from the top 1000 significantly differentially expressed genes (*p*-adj<0.01) for females and males that were upregulated or downregulated between 6 and 12 h of Tg treatment. Gene ratio is calculated as k/n, where k is the number of genes identified in each Reactome pathway, and n is the number of genes from the submitted gene list participating in any Reactome pathway. (D) Protein abundance from proteomics data of female and male mouse islets treated with DMSO or 1 μM Tg for 6 h. Top 45 differentially expressed proteins are shown (p < 0.05).

To gain insight into the differential ER stress-associated phenotypes in male and female islets, we investigated global transcriptional changes after either a 6- or 12-hour Tg treatment in each sex. Principal component analysis and unsupervised clustering shows that islets clustered by sex, treatment, and treatment time (Figure 5A; Fig. S12A). The majority of the variance was explained by treatment (Figure 5B), and pathway enrichment analysis confirms the UPR as the top upregulated pathway in Tg-treated male and female islets at both 6- and 12-hours after treatment (Figs. S13A,B; Supplementary file 4). While some UPR-associated genes differentially regulated by Tg treatment were shared between the sexes (6-hour: 29/36, 12-hour: 25/31), biological sex explained a large proportion of variance in the gene expression response to ER stress. This suggests the transcriptional response to ER stress was not fully shared between the sexes. Indeed, after a 6-hour Tg treatment, 32.6% (2247/4655) of genes that were differentially expressed between DMSO and Tg were unique to one sex (881 to females, 1376 to males). After a 12-hour Tg treatment, 29% (2259/7785) were unique to one sex (1017 to males, 1242 to females).

To describe the transcriptional response of each sex to Tg treatment in more detail, we used a two-way ANOVA to identify genes that were upregulated, downregulated, or unchanged in male and female islets between 6- and 12-hours post-Tg (Supplementary file 5). By performing pathway enrichment analysis, we were able to determine which processes were shared, and which processes differed, between the sexes during Tg treatment. For example, we observed a significant increase in mRNA levels of genes corresponding to pathways such as cellular responses to stimuli, stress, and starvation in both male and female islets between 6- and 12-hour Tg treatments (Figure 5C; Supplementary file 4), suggesting Tg has similar effects on genes related to these pathways in both sexes. Similarly, the Tg-induced changes in mRNA levels of genes related to apoptosis were largely shared between the sexes (Figs. S14A,B). While this suggests that the sex-specific regulation of genes related to apoptosis does not fully account for the susceptibility of male islets to low-dose Tg-induced cell death, in-depth studies of β cell apoptosis will be needed to confirm this point.

In contrast to these non-sex-specific changes in gene expression, there was a male-specific increase in mRNA levels of genes associated with translation during Tg treatment (Figure 5C; Supplementary file 4). In females, there was a decrease in mRNA levels of genes associated with β cell identity, such as *Pklr*, *Rfx6*, *Hnf4a*, *Slc2a2*, *Pdx1*, and *MafA* (Fig. S15A), and in genes linked with regulation of gene expression in β cells (Figure 5C). Neither of these categories were altered between 6- and 12-hour Tg treatments in males (Figure 5C; Fig. S15B). While our data suggests some aspects of the gene expression response to ER stress were shared between the sexes, we found that many genes corresponding to important cellular processes were differentially regulated during Tg treatment in only one sex.

Beyond sex-specific transcriptional changes following Tg treatment, ER stress also had a sex-specific effect on the islet proteome. Although the majority of proteins were downregulated by Tg treatment due to the generalized repression of protein synthesis under ER stress (Figure 5D), we identified 47 proteins (35 downregulated, 12 upregulated in Tg) that were differentially expressed in female islets and 82 proteins (72 downregulated, 10 upregulated in Tg) that were differentially expressed after Tg treatment in male islets (Supplementary Table 1). Proteins downregulated only in females include proteins associated with the GO term ‘endoplasmic reticulum to Golgi vesicle-mediated transport’ (GO:0006888) (BCAP31, COG5, COG3, GOSR1), whereas proteins downregulated only in males include proteins associated with GO terms ‘insulin secretion’ (GO:0030073) (PTPRN2, CLTRN, PTPRN) and ‘lysosome pathway’ (KEGG) (NPC2, CTSZ, LAMP2, PSAP, CLTA). Importantly, only seven differentially expressed proteins were in common between the sexes (Figure 5D). This suggests that as with our phenotypic and transcriptomic data, the proteomic response to Tg treatment was largely not shared between the sexes.

To integrate our islet transcriptome and partial islet proteome data, we assessed the direction of changes to mRNA and protein levels following Tg treatment. In females, the number of differentially expressed islet proteins with concordant mRNA changes was 43% (20/47), whereas the number of differentially expressed islet proteins in males with concordant mRNA changes was 49% (40/82) (Fig. S16; Supplementary file 6). This data suggests that many genes with differential mRNA expression during Tg treatment show congruent changes in protein abundance. When we next asked whether the islet genes with concordant changes in mRNA and protein levels during Tg treatment were shared between the sexes, we found that only 7% (4/56) of these genes were differentially expressed in both sexes during Tg treatment (*Tmem27*, *Emb*, *Fkbp9*, *Pdcd4*). The remaining 93% (51/55) of islet genes with concordant changes in mRNA and protein levels during Tg treatment were differentially expressed in only one sex. Thus, when taken together, our islet transcriptome and partial proteome data suggest that male and female islets show distinct responses to ER stress.

Of the islet genes with concordant changes in mRNA and protein levels, several have been linked with β cell and/or islet function. For example, Tmem27 plays a role in enhancing GSIS [94], and Pdcd4 expression is associated with β cell death under stressed conditions [95,96]. Atp6ap2 and Lamp2 have important roles in autophagy [[97], [98], [99]], Ptprn2 is required for the accumulation of insulin granules [100], and both Ptprn2 and Chga are involved in glucose-stimulated insulin secretion [101,102]. Chga is also important for maintaining islet volume and islet cell composition [103], and studies show that Tbc1d1 influences insulin secretion and β cell mass in rodents [104,105]. Because the Tbc1d1 study [104,105], and others [[95], [96], [97], [98], [99], [100], [101], [102], [103], [104]], used single- or mixed-sex models, or cell lines, future studies will need to address whether these effects on β cell and/or islet function are shared between the sexes. Additional studies will also be needed on genes identified in our analysis that were not previously linked with β cell and/or islet function (Supplementary file 6). This will elucidate whether the sex-specific regulation of mRNA and protein levels during Tg treatment affects β cell and/or islet function in either sex.

## 4 Discussion

Emerging evidence shows biological sex affects many aspects of β cell gene expression and function. Yet, many studies on β cells do not include both sexes, or fail to analyze male and female data separately. To address this gap in knowledge, the goal of our study was to provide detailed information on sex differences in islet and β cell gene expression and function in multiple contexts. In humans, we used a large scRNAseq dataset from ND and T2D donors to reveal significant male-female differences in the magnitude of gene expression changes, and in the identity of genes that were differentially regulated, between ND and T2D donors. While these data do not address the reasons for the sex-biased risk of T2D, our findings suggest β cell gene expression changes in T2D are not fully shared between the sexes. These data provide a useful resource to support future studies on how medication, disease progression, age, or body mass index contribute to the sex difference in β cell gene expression in T2D. Sex-based analysis of human β cell gene expression data will also clarify the mechanisms underlying our finding that β cells from female donors with T2D maintain higher insulin production than male donors with T2D.

In mice, we generated a large RNAseq dataset using islets isolated from 20-week-old males and females. We used an ITT to show that male and female mice have similar insulin sensitivity at this age. We acknowledge that the ITT may not be sensitive enough to pick up small differences, so future studies will need to use a hyperinsulinemic-euglycemic clamp to further compare insulin sensitivity between the sexes. Despite this potential limitation, our unbiased analysis of gene expression in islets from males and females revealed sex differences in genes associated with the UPR under normal physiological conditions. This differential gene expression was significant, as female islets were more resilient to phenotypes caused by ER stress and UPR activation than male islets, showed sex-specific transcriptional and proteomic changes in this context, and had a greater ability to maintain glucose-stimulated insulin production and secretion during ER stress. Collectively, these data suggest that in rodents, β cells from females are more resilient to ER stress. Considering the well-established links between ER stress and T2D [90,[107], [108], [109]], our data suggests a model in which female β cells have a greater ability to maintain glucose-stimulated insulin secretion in T2D because they are more resilient to ER stress and UPR activation. While future studies are needed to test this working model, and to assess the relative contribution of sex differences in β cells to the sex-biased risk of T2D, our findings highlight the importance of including both sexes in islet and β cell studies.

Including both sexes in our analysis of β cell gene expression in human ND and T2D allowed us to uncover genes that were differentially regulated in T2D in each sex. Because many of these genes may have been missed if the scRNAseq data was not analyzed by sex, our findings advance knowledge of β cell changes in T2D by identifying additional genes that are differentially regulated in this context. This knowledge adds to a growing number of studies that identify sex differences in β cell gene expression during aging in humans [12], and in mice fed either a normal [4,11] or a high fat diet [11]. Further, given that our RNAseq on islets from male and female mice with similar insulin sensitivity identifies genes and biological pathways that align with previous studies on sex differences in murine β cell gene expression [4,11], our data suggests that sex differences in islet and β cell gene expression cannot be explained solely by a male-female difference in peripheral insulin resistance. Instead, there is likely a basal sex difference in β cell gene expression that forms the foundation for sex-specific transcriptional responses to perturbations such as ER stress and T2D. By generating large islet gene expression datasets from male and female mice with similar peripheral insulin sensitivity and from islets subjected to pharmacological induction of ER stress, our studies provide a foundation of knowledge for future studies aimed at studying the causes and consequences of sex differences in islet ER stress responses and β cell function following UPR activation. This will provide deeper mechanistic insight into the sex-specific phenotypic effects reported in animal models of β cell dysfunction [[35], [36], [37], [38], [39],[110], [111], [112], [113]] and the sex-biased risk of diseases such as T2D that are associated with β cell dysfunction [12,22,113,114].

Beyond gene expression, our sex-based analysis of mouse islets allowed us to uncover male-female differences in ER stress-associated phenotypes (*e.g*. protein synthesis repression, cell death). While previous studies identify a sex difference in β cell loss in diabetic mouse models [37,39,115], and show that estrogen plays a protective role via estrogen receptor α (ERα) against ER stress to preserve β cell mass and prevent apoptosis in cell lines, mouse models, and human islets [39,115,116], we extend prior findings by showing that differences in ER stress-induced cell death were present in the context of similar insulin sensitivity between the sexes. This suggests sex differences in ER stress-associated phenotypes do not solely depend on male-female differences in peripheral insulin sensitivity. Indeed, islets isolated from males and females with similar insulin sensitivity also show a sex difference in protein synthesis repression, a classical ER stress-associated phenotype [86]. While estrogen affects insulin biosynthesis via ERα [117], future studies will need to determine whether estrogen also allows female islets to restore protein synthesis to basal levels faster than male islets following ER stress. We currently lack this knowledge, as most studies on UPR-mediated recovery from protein translation repression use single- and mixed-sex animal groups, or cultured cells [[119], [120], [121], [122], [123], [124]].

Assessing whether the recovery of protein synthesis contributes to reduced cell death in female islets following ER stress will also be an important task for future studies, as prior work suggests the inability to recover from protein synthesis repression increases ER-stress induced apoptosis [118]. Ideally, this type of study would also monitor the activity of pathways known to regulate protein synthesis repression during ER stress. For example, while we did not detect any changes in levels of phosphorylated eIF2α (also known as Eif2s1), which is known to mediate UPR-induced protein synthesis repression [86], our chosen timepoints did not overlap with the rapid changes in phospho-eIF2α following ER stress published in other studies [124,125]. A more detailed time course will therefore be necessary to assess p-eIF2α levels during ER stress in both sexes, and to test a role for phospho-eIF2α in mediating differences in protein synthesis repression. More work will also be needed to determine whether males and females differ in β cell replication [126], another ER stress-related phenotype. Ultimately, a better understanding of sex differences in ER stress-associated phenotypes in β cells will provide a mechanistic explanation for the strongly male-biased onset of diabetes-like phenotypes in mouse models of β cell ER stress (*e.g*. Akita, KINGS, Munich mice) [37,38,109]. Given the known relationship between ER stress, β cell death, and T2D, studies on the male-female difference in β cell ER stress-associated phenotypes may also advance our understanding of the male-biased risk of developing T2D in some population groups.

A further benefit of additional studies on the sex difference in β cell ER stress responses will be to identify mechanisms that support β cell insulin production. In rodents, we found that female islets maintained high glucose-stimulated insulin secretion and increased insulin content following ER stress, whereas male islets showed significant repression of high glucose-stimulated insulin secretion under the same conditions. In humans, while a study using a mixed-sex group of T2D donors shows β cells experience ER stress associated with β cell dysfunction [63], we found that changes to β cell insulin secretion in T2D were not the same between the sexes. Specifically, the magnitude of the reduction in insulin release by β cells from female donors with T2D was smaller than in β cells from male donors with T2D. Together with our data from rodents, this suggests female β cells maintain enhanced insulin production and/or secretion in multiple contexts, and the increased β cell function cannot be solely attributed to a sex difference in peripheral insulin sensitivity.

Clues into potential ways that female β cells maintain improved insulin production and secretion emerge from our examination of the transcriptional response to ER stress in mice of each sex. Our data shows that Tg treatment induces gene expression changes characteristic of ER stress [127], and revealed similar biological pathways that were upregulated in T2D donors. Furthermore, we identified significant differences between male and female islets in the transcriptional response to ER stress over time. One notable finding was that a greater number of β cell identity genes were downregulated between 6- and 12-hour Tg treatments in females, but not in males. Because most studies on the relationship between β cell identity and function used a mixed-sex pool of islets and β cells [79,128,129], more studies will be needed to test whether there are sex-specific changes to β cell identity during ER stress, and to determine the functional consequences of this sex-specific effect.

Overall, our data demonstrates sex differences in β cell function in multiple contexts. One potential explanation for these differences is the sex-specific regulation of β cell ER stress responses and function. Indeed, sex differences in ER stress and protein markers of apoptosis were observed in mouse kidney cells [130], suggesting that studying β cell ER stress may provide insight into this difference in other cell types. Alternatively, it is possible that there are sex differences in β cell number that account for the male-female differences in β cell function that we observe. For example, considering two published studies indicate males have fewer β cells [6,37], the burden of maintaining glucose homeostasis may fall on a smaller number of cells in males, leading to higher susceptibility to ER stress. Future studies will need to address sex differences in β cell number relative to pancreas size and body size to test this possibility. Ultimately, a better understanding of changes to β cell gene expression and function in males and females will suggest effective ways to reverse disease-associated changes to this important cell type in each sex, improving equity in health outcomes [131].

### 4.1 Conclusions

Our study reports significant sex differences in islet and β cell gene expression and stress responses in both humans and mice. These differences likely contribute to sex differences in β cell resilience, allowing female β cells to show a greater ability to maintain glucose-stimulated insulin production and secretion across multiple contexts. This knowledge forms a foundation for future studies aimed at understanding how sex differences in β cell function affect physiology and the pathophysiology of diseases such as T2D.

## Author contributions

G. P. B. conceived studies, conducted experiments, interpreted experiments, wrote the manuscript

Y. X. performed bioinformatic analysis and data visualization

J. C. created custom R scripts (single-cell GFP tracking)

S. W. analyzed data (human RNAseq)

C. C. created custom R scripts (mouse RNAseq analysis)

J. A. Z. analyzed data (HPAP perifusions)

S. S. conducted experiments (*in vivo* physiology)

E. P. conducted experiments (islet western blots)

X. H. conducted experiments (dissections)

J. D. J. conceived studies, interpreted experiments, edited the manuscript

E. J. R. conceived studies, interpreted experiments, edited the manuscript, and is the guarantor of this work

## Funding

This study was supported by operating grants to E.J.R. from the 10.13039/501100000245Michael Smith Foundation for Health Research (16876), 10.13039/501100000024Canadian Institutes of Health Research (GS4-171365), the Canadian Foundation for Innovation (JELF-34879), and Diabetes Canada (OG-3-22-5646-ER), and to J.D.J. (PJT-152999) from the 10.13039/501100000024Canadian Institutes of Health Research, and core support from the 10.13039/100009881JDRF Centre of Excellence at 10.13039/501100005247UBC (3-COE-2022-1103-M-B). J.D.J. was funded by a Diabetes Investigator Award from 10.13039/100013528Diabetes Canada.

## Data Availability

Details of all statistical tests and p-values as well as the raw data are provided in Supplementary files. Supplementary files are available as Brownrigg, George (2023), “Sex differences in islet stress responses support female β cell resilience”, Mendeley Data, V1, doi: https://doi.org/10.17632/ftcs6xj9ft.1. RNAseq data is available at PRJNA842443 and PRJNA842371.

## References

[1] Parchami A., Kusha S. (2015). Effect of sex on histomorphometric properties of Langerhans islets in native chickens. Vet Res Forum.

[2] Rideout E.J., Narsaiya M.S., Grewal S.S. (2015 Dec 28). The sex determination gene transformer regulates male-female differences in Drosophila body size. PLoS Genet.

[3] Millington J.W., Brownrigg G.P., Chao C., Sun Z., Basner-Collins P.J., Wat L.W. (2021 Jan 15). Female-biased upregulation of insulin pathway activity mediates the sex difference in Drosophila body size plasticity. Elife.

[4] Stancill J.S., Osipovich A.B., Cartailler J.P., Magnuson M.A. (2019 Feb 1). Transgene-associated human growth hormone expression in pancreatic β-cells impairs identification of sex-based gene expression differences. Am J Physiol Endocrinol Metab.

[5] Horie I., Abiru N., Eto M., Sako A., Akeshima J., Nakao T. (2018 Nov). Sex differences in insulin and glucagon responses for glucose homeostasis in young healthy Japanese adults. J Diabetes Investig.

[6] Marchese E., Rodeghier C., Monson R.S., McCracken B., Shi T., Schrock W. (2015 Nov 1). Enumerating β-cells in whole human islets: sex differences and associations with clinical outcomes after islet transplantation. Diabetes Care.

[7] Oliva M., Muñoz-Aguirre M., Kim-Hellmuth S., Wucher V., Gewirtz A.D.H., Cotter D.J. (2020 Sep 11). The impact of sex on gene expression across human tissues. Science.

[8] Enge M., Arda H.E., Mignardi M., Beausang J., Bottino R., Kim S.K. (2017 Oct 5). Single-cell analysis of human pancreas reveals transcriptional signatures of aging and somatic mutation patterns. Cell.

[9] Schaum N., Lehallier B., Hahn O., Pálovics R., Hosseinzadeh S., Lee S.E. (2020 Jul). Aging hallmarks exhibit organ-specific temporal signatures. Nature.

[10] Hall E., Volkov P., Dayeh T., Esguerra J.L.S., Salö S., Eliasson L. (2014 Dec 3). Sex differences in the genome-wide DNA methylation pattern and impact on gene expression, microRNA levels and insulin secretion in human pancreatic islets. Genome Biol.

[11] Liu G., Li Y., Zhang T., Li M., Li S., He Q. (2021 Jun 1). Single-cell RNA sequencing reveals sexually dimorphic transcriptome and type 2 diabetes genes in mouse islet β cells. Dev Reprod Biol.

[12] Arrojoe Drigo R., Erikson G., Tyagi S., Capitanio J., Lyon J., Spigelman A.F. (2019 Aug). Aging of human endocrine pancreatic cell types is heterogeneous and sex-specific. bioRxiv.

[13] Li T., Jiao W., Li W., Li H. (2016 Jul 2). Sex effect on insulin secretion and mitochondrial function in pancreatic beta cells of elderly Wistar rats. Endocr Res.

[14] Tura A., Pacini G., Moro E., Vrbíková J., Bendlová B., Kautzky-Willer A. (2018 Dec 1). Sex- and age-related differences of metabolic parameters in impaired glucose metabolism and type 2 diabetes compared to normal glucose tolerance. Diabetes Res Clin Pract.

[15] Kautzky-Willer A., Brazzale A.R., Moro E., Vrbíková J., Bendlova B., Sbrignadello S. (2012). Influence of increasing BMI on insulin sensitivity and secretion in normotolerant men and women of a wide age span. Obesity.

[16] Basu R., Man C.D., Campioni M., Basu A., Klee G., Toffolo G. (2006 Jul 1). Effects of age and sex on postprandial glucose metabolism: differences in glucose turnover, insulin secretion, insulin action, and hepatic insulin extraction. Diabetes.

[17] Nuutila P., Knuuti M.J., Mäki M., Laine H., Ruotsalainen U., Teräs M. (1995 Jan 1). Gender and insulin sensitivity in the heart and in skeletal muscles: studies using positron emission tomography. Diabetes.

[18] Lundsgaard A.M., Kiens B. (2014 Nov 13). Gender differences in skeletal muscle substrate metabolism – molecular mechanisms and insulin sensitivity. Front Endocrinol.

[19] Borissova A.M., Tankova T., Kirilov G., Koev D. (2005). Gender-dependent effect of ageing on peripheral insulin action. Int J Clin Pract.

[20] Geer E.B., Shen W. (2009 Jan 1). Gender differences in insulin resistance, body composition, and energy balance. Gend Med.

[21] Færch K., Borch-Johnsen K., Vaag A., Jørgensen T., Witte D.R. (2010 May 1). Sex differences in glucose levels: a consequence of physiology or methodological convenience? The Inter99 study. Diabetologia.

[22] Gannon M., Kulkarni R.N., Tse H.M., Mauvais-Jarvis F. (2018 Sep). Sex differences underlying pancreatic islet biology and its dysfunction. Mol Metabol.

[23] Tramunt B., Smati S., Grandgeorge N., Lenfant F., Arnal J.F., Montagner A. (2019 Nov 21). Sex differences in metabolic regulation and diabetes susceptibility. Diabetologia.

[24] Macotela Y., Boucher J., Tran T.T., Kahn C.R. (2009 Apr). Sex and depot differences in adipocyte insulin sensitivity and glucose metabolism. Diabetes.

[25] Rudnicki M, Abdifarkosh G, Rezvan O, Nwadozi E, Roudier E, Haas TL (2018). Female Mice Have Higher Angiogenesis in Perigonadal Adipose Tissue Than Males in Response to High-Fat Diet. Front Physiol.

[26] Millington J.W., Biswas P., Chao C., Xia Y.H., Wat L.W., Brownrigg G.P. (2022 Feb 23). A low sugar diet enhances Drosophila body size in males and females via sex-specific mechanisms. Development.

[27] Mank J.E., Rideout E.J. (2021 Oct 14). Developmental mechanisms of sex differences: from cells to organisms. Development.

[28] Oster R.T., Johnson J.A., Hemmelgarn B.R., King M., Balko S.U., Svenson L.W. (2011 Sep 6). Recent epidemiologic trends of diabetes mellitus among status Aboriginal adults. CMAJ (Can Med Assoc J).

[29] Dyck R., Osgood N., Lin T.H., Gao A., Stang M.R. (2010 Feb 23). Epidemiology of diabetes mellitus among First Nations and non-First Nations adults. CMAJ (Can Med Assoc J).

[30] Zhou B., Lu Y., Hajifathalian K., Bentham J., Cesare M.D., Danaei G. (2016 Apr 9). Worldwide trends in diabetes since 1980: a pooled analysis of 751 population-based studies with 4·4 million participants. Lancet.

[31] Kautzky-Willer A., Harreiter J., Pacini G. (2016 Jun). Sex and gender differences in risk, pathophysiology and complications of type 2 diabetes mellitus. Endocr Rev.

[32] Heise L., Greene M.E., Opper N., Stavropoulou M., Harper C., Nascimento M. (2019 Jun 15). Gender inequality and restrictive gender norms: framing the challenges to health. Lancet.

[33] Kautzky-Willer A., Harreiter J. (2017 Sep). Sex and gender differences in therapy of type 2 diabetes. Diabetes Res Clin Pract.

[34] Corsetti J.P., Sparks J.D., Peterson R.G., Smith R.L., Sparks C.E. (2000 Feb). Effect of dietary fat on the development of non-insulin dependent diabetes mellitus in obese Zucker diabetic fatty male and female rats. Atherosclerosis.

[35] Paik S.G., Michelis M.A., Kim Y.T., Shin S. (1982 Aug). Induction of insulin-dependent diabetes by streptozotocin. Inhibition by estrogens and potentiation by androgens. Diabetes.

[36] Verchere C.B., D'Alessio D.A., Palmiter R.D., Weir G.C., Bonner-Weir S., Baskin D.G. (1996 Apr 16). Islet amyloid formation associated with hyperglycemia in transgenic mice with pancreatic beta cell expression of human islet amyloid polypeptide. Proc Natl Acad Sci U S A.

[37] Austin A.L.F., Daniels Gatward L.F., Cnop M., Santos G., Andersson D., Sharp S. (2020 Dec). The KINGS ins2+/G32S mouse: a novel model of β-cell endoplasmic reticulum stress and human diabetes. Diabetes.

[38] Yoshioka M., Kayo T., Ikeda T., Koizuni A. (1997 May 1). A novel locus, Mody4, distal to D7Mit189 on chromosome 7 determines early-onset NIDDM in nonobese C57BL/6 (akita) mutant mice. Diabetes.

[39] May C.L., Chu K., Hu M., Ortega C.S., Simpson E.R., Korach K.S. (2006 Jun 13). Estrogens protect pancreatic β-cells from apoptosis and prevent insulin-deficient diabetes mellitus in mice. Proc Natl Acad Sci U S A.

[40] Cohrs C.M., Panzer J.K., Drotar D.M., Enos S.J., Kipke N., Chen C. (2020 Apr 7). Dysfunction of persisting β cells is a key feature of early type 2 diabetes pathogenesis. Cell Rep.

[41] Saisho Y. (2015 Feb 15). β-cell dysfunction: its critical role in prevention and management of type 2 diabetes. World J Diabetes.

[42] Segerstolpe Å., Palasantza A., Eliasson P., Andersson E.M., Andréasson A.C., Sun X. (2016 Oct 11). Single-cell transcriptome profiling of human pancreatic islets in health and type 2 diabetes. Cell Metabol.

[43] Xin Y., Kim J., Okamoto H., Ni M., Wei Y., Adler C. (2016 Oct 11). RNA sequencing of single human islet cells reveals type 2 diabetes genes. Cell Metabol.

[44] Avrahami D., Wang Y.J., Schug J., Feleke E., Gao L., Liu C. (2020 Jul 30). Single-cell transcriptomics of human islet ontogeny defines the molecular basis of β-cell dedifferentiation in T2D. Mol Metabol.

[45] Lawlor N., George J., Bolisetty M., Kursawe R., Sun L., Sivakamasundari V. (2017 Feb 1). Single-cell transcriptomes identify human islet cell signatures and reveal cell-type–specific expression changes in type 2 diabetes. Genome Res.

[46] Baron M., Veres A., Wolock S.L., Faust A.L., Gaujoux R., Vetere A. (2016 Oct 26). A single-cell transcriptomic map of the human and mouse pancreas reveals inter- and intra-cell population structure. Cell Systems.

[47] Muraro M.J., Dharmadhikari G., Grün D., Groen N., Dielen T., Jansen E. (2016 Oct 26). A single-cell transcriptome atlas of the human pancreas. Cell Systems.

[48] Deng S., Vatamaniuk M., Huang X., Doliba N., Lian M.M., Frank A. (2004 Mar 1). Structural and functional abnormalities in the islets isolated from type 2 diabetic subjects. Diabetes.

[49] Wu M., Lee M.Y.Y., Bahl V., Traum D., Schug J., Kusmartseva I. (2021 Nov 2). Single-cell analysis of the human pancreas in type 2 diabetes using multi-spectral imaging mass cytometry. Cell Rep.

[50] Salvalaggio P.R.O., Deng S., Ariyan C.E., Millet I., Zawalich W.S., Basadonna G.P. (2002 Sep 27). Islet filtration: a simple and rapid new purification procedure that avoids ficoll and improves islet mass and function. Transplantation.

[51] Chu C.M.J., Modi H., Ellis C., Krentz N.A.J., Skovsø S., Zhao Y.B. (2022 Dec 1). Dynamic Ins2 gene activity defines β-cell maturity states. Diabetes.

[52] Truchan N.A., Brar H.K., Gallagher S.J., Neuman J.C., Kimple M.E. (2015 Oct 9). A single-islet microplate assay to measure mouse and human islet insulin secretion. Islets.

[53] Skovsø S., Panzhinskiy E., Kolic J., Cen H.H., Dionne D.A., Dai X.Q. (2022 Feb 8). Beta-cell specific Insr deletion promotes insulin hypersecretion and improves glucose tolerance prior to global insulin resistance. Nat Commun.

[54] Szabat M., Page M.M., Panzhinskiy E., Skovsø S., Mojibian M., Fernandez-Tajes J. (2016 Jan 12). Reduced insulin production relieves endoplasmic reticulum stress and induces β cell proliferation. Cell Metabol.

[55] Dobin A., Davis C.A., Schlesinger F., Drenkow J., Zaleski C., Jha S. (2013 Jan 1). STAR: ultrafast universal RNA-seq aligner. Bioinformatics.

[56] Love M.I., Huber W., Anders S. (2014 Dec 5). Moderated estimation of fold change and dispersion for RNA-seq data with DESeq2. Genome Biol.

[57] Gillespie M., Jassal B., Stephan R., Milacic M., Rothfels K., Senff-Ribeiro A. (2022 Jan 7). The reactome pathway knowledgebase 2022. Nucleic Acids Res.

[58] Zhou G., Soufan O., Ewald J., Hancock R.E.W., Basu N., Xia J. (2019 Jul 2). NetworkAnalyst 3.0: a visual analytics platform for comprehensive gene expression profiling and meta-analysis. Nucleic Acids Res.

[59] Tyanova S., Temu T., Sinitcyn P., Carlson A., Hein M.Y., Geiger T. (2016 Sep). The Perseus computational platform for comprehensive analysis of (prote)omics data. Nat Methods.

[60] Wei T., Viliam S. (2021). https://taiyun.github.io/corrplot/.

[61] Wang S., Flibotte S., Camunas-Soler J., MacDonald P.E., Johnson J.D. (2021 Dec 1). A new hypothesis for type 1 diabetes risk: the at-risk allele at rs3842753 associates with increased beta-cell INS messenger RNA in a meta-analysis of single-cell RNA-sequencing data. Can J Diabetes.

[62] Hashemitabar M., Bahramzadeh S., Saremy S., Nejaddehbashi F. (2015 Sep). Glucose plus metformin compared with glucose alone on β-cell function in mouse pancreatic islets. Biomed Rep.

[63] Marchetti P., Bugliani M., Lupi R., Marselli L., Masini M., Boggi U. (2007 Dec 1). The endoplasmic reticulum in pancreatic beta cells of type 2 diabetes patients. Diabetologia.

[64] Lupi R., Del Guerra S., Fierabracci V., Marselli L., Novelli M., Patanè G. (2002 Feb). Lipotoxicity in human pancreatic islets and the protective effect of metformin. Diabetes.

[65] Kanda Y., Shimoda M., Hamamoto S., Tawaramoto K., Kawasaki F., Hashiramoto M. (2010 Feb). Molecular mechanism by which pioglitazone preserves pancreatic β-cells in obese diabetic mice: evidence for acute and chronic actions as a PPARγ agonist. Am J Physiol Endocrinol Metab.

[66] Murao N., Yokoi N., Takahashi H., Hayami T., Minami Y., Seino S. (2022 Jan). Increased glycolysis affects β-cell function and identity in aging and diabetes. Mol Metabol.

[67] Mizukami H., Takahashi K., Inaba W., Osonoi S., Kamata K., Tsuboi K. (2014 Feb 12). Age-associated changes of islet endocrine cells and the effects of body mass index in Japanese. J Diabetes Investig.

[68] Yinhui H.E., Haiyan X.U., Qi F.U., Tao Y. (2019 Sep 30). Effects of glycosylated hemoglobin and disease course on islet β-cell function in patients with type 2 diabetes. Nan Fang Yi Ke Da Xue Xue Bao.

[69] Kaestner K.H., Powers A.C., Naji A., Consortium H.P.A.P., Atkinson M.A. (2019 Jul). NIH initiative to improve understanding of the pancreas, islet, and autoimmunity in type 1 diabetes: the human pancreas analysis Program (HPAP). Diabetes.

[70] Hellman B., Idahl L.Å., Lernmark Å., Täljedal I.B. (1974 Sep). The pancreatic β-cell recognition of insulin secretagogues: does cyclic AMP mediate the effect of glucose?. Proc Natl Acad Sci USA.

[71] Yang X., Schadt E.E., Wang S., Wang H., Arnold A.P., Ingram-Drake L. (2006 Aug 1). Tissue-specific expression and regulation of sexually dimorphic genes in mice. Genome Res.

[72] Lu T., Mar J.C. (2020 Nov 5). Investigating transcriptome-wide sex dimorphism by multi-level analysis of single-cell RNA sequencing data in ten mouse cell types. Biol Sex Differ.

[73] Cottet-Dumoulin D., Lavallard V., Lebreton F., Wassmer C.H., Bellofatto K., Parnaud G. (2020 Dec 25). Biosynthetic activity differs between islet cell types and in beta cells is modulated by glucose and not by secretion. Endocrinology.

[74] Li N., Yang Z., Li Q., Yu Z., Chen X., Li J.C. (2018 Jun 7). Ablation of somatostatin cells leads to impaired pancreatic islet function and neonatal death in rodents. Cell Death Dis.

[75] Webb G.C., Dey A., Wang J., Stein J., Milewski M., Steiner D.F. (2004 Jul 23). Altered proglucagon processing in an α-cell line derived from prohormone convertase 2 null mouse islets. J Biol Chem.

[76] Garcia-Barrado M.J., Ravier M.A., Rolland J.F., Gilon P., Nenquin M., Henquin J.C. (2001 Jan 1). Inhibition of protein synthesis sequentially impairs distinct steps of stimulus-secretion coupling in pancreatic β cells. Endocrinology.

[77] Scheuner D., Mierde D.V., Song B., Flamez D., Creemers J.W.M., Tsukamoto K. (2005 Jul). Control of mRNA translation preserves endoplasmic reticulum function in beta cells and maintains glucose homeostasis. Nat Med.

[78] Izumi T., Yokota-Hashimoto H., Zhao S., Wang J., Halban P.A., Takeuchi T. (2003 Feb). Dominant negative pathogenesis by mutant proinsulin in the Akita diabetic mouse. Diabetes.

[79] Xin Y., Dominguez Gutierrez G., Okamoto H., Kim J., Lee A.H., Adler C. (2018). Pseudotime ordering of single human β-cells reveals states of insulin production and unfolded protein response. Diabetes.

[80] Lipson K.L., Fonseca S.G., Ishigaki S., Nguyen L.X., Foss E., Bortell R. (2006 Sep). Regulation of insulin biosynthesis in pancreatic beta cells by an endoplasmic reticulum-resident protein kinase IRE1. Cell Metabol.

[81] Teodoro T., Odisho T., Sidorova E., Volchuk A. (2012 Apr). Pancreatic β-cells depend on basal expression of active ATF6α-p50 for cell survival even under nonstress conditions. Am J Physiol Cell Physiol.

[82] Scheuner D., Song B., McEwen E., Liu C., Laybutt R., Gillespie P. (2001 Jun). Translational control is required for the unfolded protein response and in vivo glucose homeostasis. Mol Cell.

[83] Luciani D.S., Gwiazda K.S., Yang T.L.B., Kalynyak T.B., Bychkivska Y., Frey M.H.Z. (2009 Feb 1). Roles of IP3R and RyR Ca2+ channels in endoplasmic reticulum stress and β-cell death. Diabetes.

[84] Riggs A.C., Bernal-Mizrachi E., Ohsugi M., Wasson J., Fatrai S., Welling C. (2005 Nov 1). Mice conditionally lacking the Wolfram gene in pancreatic islet beta cells exhibit diabetes as a result of enhanced endoplasmic reticulum stress and apoptosis. Diabetologia.

[85] Li J., Lee B., Lee A.S. (2006 Mar 17). Endoplasmic reticulum stress-induced apoptosis: multiple pathways and activation of p53-up-regulated modulator of apoptosis (PUMA) and NOXA by p53. J Biol Chem.

[86] Sharma R.B., Landa-Galván H.V., Alonso L.C. (2021 Nov). Living dangerously: protective and harmful ER stress responses in pancreatic β-cells. Diabetes.

[87] Gwiazda K.S., Yang T.L.B., Lin Y., Johnson J.D. (2009 Apr). Effects of palmitate on ER and cytosolic Ca2+ homeostasis in β-cells. Am J Physiol Endocrinol Metab.

[88] Fonseca S.G., Gromada J., Urano F. (2011 Jul). Endoplasmic reticulum stress and pancreatic beta cell death. Trends Endocrinol Metabol.

[89] Wakae-Takada N., Xuan S., Watanabe K., Meda P., Leibel R.L. (2013 Apr). Molecular basis for the regulation of islet beta cell mass in mice: the role of E-cadherin. Diabetologia.

[90] Shrestha N., Franco E.D., Arvan P., Cnop M. (2021). Pathological β-cell endoplasmic reticulum stress in type 2 diabetes: current evidence. Front Endocrinol.

[91] Oyadomari S., Mori M. (2004 Apr). Roles of CHOP/GADD153 in endoplasmic reticulum stress. Cell Death Differ.

[92] Li T., Jiao W., Li W., Li H. (2016 Aug). Sex effect on insulin secretion and mitochondrial function in pancreatic beta cells of elderly Wistar rats. Endocr Res.

[93] Basu A., Dube S., Basu R., Mauvais-Jarvis F. (2017). Sex and gender factors affecting metabolic homeostasis, diabetes and obesity [Internet].

[94] Altirriba J., Gasa R., Casas S., Ramírez-Bajo M.J., Ros S., Gutierrez-Dalmau A. (2010 Jul 1). The role of transmembrane protein 27 (TMEM27) in islet physiology and its potential use as a beta cell mass biomarker. Diabetologia.

[95] Kumar S., Marriott C.E., Alhasawi N.F., Bone A.J., Macfarlane W.M. (2017 Jul 27). The role of tumour suppressor PDCD4 in beta cell death in hypoxia. PLoS One.

[96] Ruan Q., Wang T., Kameswaran V., Wei Q., Johnson D.S., Matschinsky F. (2011 Jul 19). The microRNA-21−PDCD4 axis prevents type 1 diabetes by blocking pancreatic β cell death. Proc Natl Acad Sci U S A.

[97] Binger K.J., Neukam M., Tattikota S.G., Qadri F., Puchkov D., Willmes D.M. (2019 Oct). Atp6ap2 deletion causes extensive vacuolation that consumes the insulin content of pancreatic β cells. Proc Natl Acad Sci USA.

[98] Masini M., Bugliani M., Lupi R., del Guerra S., Boggi U., Filipponi F. (2009 Jun 1). Autophagy in human type 2 diabetes pancreatic beta cells. Diabetologia.

[99] Ji J., Petropavlovskaia M., Khatchadourian A., Patapas J., Makhlin J., Rosenberg L. (2019 Apr). Type 2 diabetes is associated with suppression of autophagy and lipid accumulation in β-cells. J Cell Mol Med.

[100] Cai T., Hirai H., Zhang G., Zhang M., Takahashi N., Kasai H. (2011 Sep). Deletion of Ia-2 and/or Ia-2β in mice decreases insulin secretion by reducing the number of dense core vesicles. Diabetologia.

[101] Torii S., Kubota C., Saito N., Kawano A., Hou N., Kobayashi M. (2018 Apr 20). The pseudophosphatase phogrin enables glucose-stimulated insulin signaling in pancreatic β cells. J Biol Chem.

[102] Wollam J., Mahata S., Riopel M., Hernandez-Carretero A., Biswas A., Bandyopadhyay G.K. (2017 Jun). Chromogranin A regulates vesicle storage and mitochondrial dynamics to influence insulin secretion. Cell Tissue Res.

[103] Portela-Gomes G.M., Gayen J.R., Grimelius L., Stridsberg M., Mahata S.K. (2008 Nov 29). The importance of chromogranin A in the development and function of endocrine pancreas. Regul Pept.

[104] Paglialunga S., Simnett G., Robson H., Hoang M., Pillai R., Arkell A.M. (2017 Jun). The Rab-GTPase activating protein, TBC1D1, is critical for maintaining normal glucose homeostasis and β-cell mass. Appl Physiol Nutr Metabol.

[105] Stermann T., Menzel F., Weidlich C., Jeruschke K., Weiss J., Altenhofen D. (2018 Apr 1). Deletion of the RabGAP TBC1D1 leads to enhanced insulin secretion and fatty acid oxidation in islets from male mice. Endocrinology.

[106] Laybutt D.R., Preston A.M., Åkerfeldt M.C., Kench J.G., Busch A.K., Biankin A.V. (2007 Apr 1). Endoplasmic reticulum stress contributes to beta cell apoptosis in type 2 diabetes. Diabetologia.

[107] Engin F., Nguyen T., Yermalovich A., Hotamisligil G.S. (2014 Feb 11). Aberrant islet unfolded protein response in type 2 diabetes. Sci Rep.

[108] jiang Huang C., yu Lin C., Haataja L., Gurlo T., Butler A.E., Rizza R.A. (2007 Aug). High expression rates of human islet amyloid polypeptide induce endoplasmic reticulum stress mediated beta-cell apoptosis, a characteristic of humans with type 2 but not type 1 diabetes. Diabetes.

[109] Herbach N., Rathkolb B., Kemter E., Pichl L., Klaften M., de Angelis M.H. (2007 May 1). Dominant-negative effects of a novel mutated Ins2 allele causes early-onset diabetes and severe β-cell loss in Munich Ins2C95S mutant mice. Diabetes.

[110] Tiano J.P., Mauvais-Jarvis F. (2012 Jun). Importance of oestrogen receptors to preserve functional β-cell mass in diabetes. Nat Rev Endocrinol.

[111] Janson J., Soeller W.C., Roche P.C., Nelson R.T., Torchia A.J., Kreutter D.K. (1996 Jul 9). Spontaneous diabetes mellitus in transgenic mice expressing human islet amyloid polypeptide. Proc Natl Acad Sci U S A.

[112] Walker E.M., Cha J., Tong X., Guo M., Liu J.H., Yu S. (2021 Oct 12). Sex-biased islet β cell dysfunction is caused by the MODY MAFA S64F variant by inducing premature aging and senescence in males. Cell Rep.

[113] Mauvais-Jarvis F., Sobngwi E., Porcher R., Riveline J.P., Kevorkian J.P., Vaisse C. (2004 Mar 1). Ketosis-prone type 2 diabetes in patients of sub-saharan african origin: clinical pathophysiology and natural history of β-cell dysfunction and insulin resistance. Diabetes.

[114] Iacovazzo D., Flanagan S.E., Walker E., Quezado R., de Sousa Barros F.A., Caswell R. (2018 Jan 30). MAFA missense mutation causes familial insulinomatosis and diabetes mellitus. Proc Natl Acad Sci U S A.

[115] Xu B., Allard C., Alvarez-Mercado A.I., Fuselier T., Kim J.H., Coons L.A. (2018 Jul 3). Estrogens promote misfolded proinsulin degradation to protect insulin production and delay diabetes. Cell Rep.

[116] Zhou Z., Ribas V., Rajbhandari P., Drew B.G., Moore T.M., Fluitt A.H. (2018 Mar 30). Estrogen receptor α protects pancreatic β-cells from apoptosis by preserving mitochondrial function and suppressing endoplasmic reticulum stress. J Biol Chem.

[117] Alonso-Magdalena P., Ropero A.B., Carrera M.P., Cederroth C.R., Baquié M., Gauthier B.R. (2008 Apr 30). Pancreatic insulin content regulation by the estrogen receptor ER alpha. PLoS One.

[118] Cnop M., Ladriere L., Hekerman P., Ortis F., Cardozo A.K., Dogusan Z. (2007 Feb 9). Selective inhibition of eukaryotic translation initiation factor 2α dephosphorylation potentiates fatty acid-induced endoplasmic reticulum stress and causes pancreatic β-cell dysfunction and apoptosis. J Biol Chem.

[119] Oyadomari S., Koizumi A., Takeda K., Gotoh T., Akira S., Araki E. (2002 Feb 15). Targeted disruption of the Chop gene delays endoplasmic reticulum stress–mediated diabetes. J Clin Invest.

[120] Han J., Back S.H., Hur J., Lin Y.H., Gildersleeve R., Shan J. (2013 May). ER-stress-induced transcriptional regulation increases protein synthesis leading to cell death. Nat Cell Biol.

[121] Harding H.P., Zeng H., Zhang Y., Jungries R., Chung P., Plesken H. (2001 Jun 1). Diabetes mellitus and exocrine pancreatic dysfunction in Perk−/− mice reveals a role for translational control in secretory cell survival. Mol Cell.

[122] Novoa I., Zhang Y., Zeng H., Jungreis R., Harding H.P., Ron D. (2003 Mar 3). Stress-induced gene expression requires programmed recovery from translational repression. EMBO J.

[123] Ma Y., Hendershot L.M. (2003 Sep 12). Delineation of a negative feedback regulatory loop that controls protein translation during endoplasmic reticulum stress. J Biol Chem.

[124] Panzhinskiy E., Skovsø S., Cen H.H., Chu K.Y., MacDonald K., Soukhatcheva G. (2021 Feb 17). Eukaryotic translation initiation factor 2A protects pancreatic beta cells during endoplasmic reticulum stress while rescuing translation inhibition. bioRxiv.

[125] Cnop M., Toivonen S., Igoillo-Esteve M., Salpea P. (2017 Jul 12). Endoplasmic reticulum stress and eIF2α phosphorylation: the Achilles heel of pancreatic β cells. Mol Metabol.

[126] Sharma R.B., O'Donnell A.C., Stamateris R.E., Ha B., McCloskey K.M., Reynolds P.R. (2015 Oct 1). Insulin demand regulates β cell number via the unfolded protein response. J Clin Invest.

[127] Sharma R.B., Darko C., Alonso L.C. (2020 Oct 9). Intersection of the ATF6 and XBP1 ER stress pathways in mouse islet cells. J Biol Chem.

[128] Nasteska D., Fine N.H.F., Ashford F.B., Cuozzo F., Viloria K., Smith G. (2021 Jan 29). PDX1LOW MAFALOW β-cells contribute to islet function and insulin release. Nat Commun.

[129] Talchai C., Xuan S., Lin H.V., Sussel L., Accili D. (2012 Sep 14). Pancreatic β-cell dedifferentiation as mechanism of diabetic β-cell failure. Cell.

[130] Hodeify R., Megyesi J., Tarcsafalvi A., Mustafa H.I., Seng N.S.H.L., Price P.M. (2013 Apr 1). Pathophysiology of Acute Kidney Injury: gender differences control the susceptibility to ER stress-induced acute kidney injury. Am J Physiol Ren Physiol.

[131] Manicardi V., Russo G., Napoli A., Torlone E., Li Volsi P., Giorda C.B. (2016 Oct 3). Gender-disparities in adults with type 1 diabetes: more than a quality of care issue. A cross-sectional observational study from the AMD annals initiative. PLoS One.

