# Supplementary material for: Sex differences in islet stress responses support female β cell resilience
Source: Mol Metab. 2023 Jan 20;69:101678. doi: 10.1016/j.molmet.2023.101678 (PMC9971554; doi:10.1016/j.molmet.2023.101678)
Supplement: Multimedia component 2 [file mmc2.docx]

**SUPPLEMENTAL FIGURE LEGENDS**

**Figure S1. Sex-specific and non-sex-specific differentially expressed genes in T2D.** scRNAseq data from male and female human β cells. (A-C) Top 60 significantly differentially expressed genes (*p*-adj < 0.05). Non-sex-specific (A), female-specific (B), or male-specific (C). For complete gene lists see Supplementary file 2.

**Figure S2. Gene expression changes in T2D.** scRNAseq data from male and female human β cells. (A, B) Top 60 differentially expressed genes (*p*-adj < 0.05) in females (A) and males (B). Sex-specific genes are indicated in red text. For complete gene lists see Supplementary file 2.

**Figure S3. Correlations between donor attributes and insulin secretion.** A Pearson correlation between human donor attributes and insulin secretion across each condition is shown. (A) donors with Type 2 diabetes, (B) all human donors. Significant correlations are denoted with a star (*), boxes are shaded according to the correlation coefficient (blue = negative correlation, red = positive correlation). For donor metadata see Supplementary file 8.

**Figure S4. Similar insulin sensitivity in male and female mice.** (A) Insulin tolerance test (ITT). 20-week-old female and male B6 mice were fasted for 6 hours. Glucose levels (% baseline) from insulin tolerance tests (ITT) following a single insulin injection (0.75U insulin/kg body weight). AUC calculations (n=11 females, n=11 males). ns indicates not significant; error bars indicate SEM.

**Figure S5. Mouse islet gene expression clusters by sex.** (A) Unsupervised hierarchical clustering of RNAseq data from female and male mouse islets. Sorting was based on all genes where the total count was >10 across all samples.

**Figure S6. ER stress-induced protein synthesis repression persists in male mouse islet cells.** (A) Representative images of dispersed islets stained with nuclear mask and OPP labeled with Alexa Fluor 594. (B, C) Integrated staining intensity of Alexa Fluor 594 in nuclear mask positive islet cells in control media (B, FBS+) or after treatment with DMSO control or 1 μM Tg for 2- or 24-hours (C, FBS-). Protein synthesis is displayed on a per cell basis from data shown in Figure 3. n=4-5 mice, >1000 cells per group. Mean values are indicated under each group. (B) Protein synthesis was significantly higher in male islet cells than female islet cells in control media, 3.9% (p=0.027; unpaired Student’s *t*-test). (C) In female islet cells, protein synthesis was significantly repressed from control-2 hour treatments (p<0.0001; unpaired Student’s *t-*test) and significantly increased from both control-24 hour treatments and 2-24 hour treatments (p<0.0001; unpaired Student’s *t-*test). In male islet cells, protein synthesis was significantly repressed from control-2 hour treatments (p<0.0001; unpaired Student’s *t*-test) and control-24 hour treatments (p<0.0001; unpaired Student’s *t-*test); however, was not significantly different between 2-24 hour treatments (p=0.07; unpaired Student’s *t*-test). * indicates p<0.05, *** indicates p<0.001, **** indicates p<0.0001; ns indicates not significant; error bars indicate SEM.

**Figure S7. *Ins2* gene activity is repressed by ER stress induction.** *Ins2* gene activity in β cells from 20-week-old male and female B6 mice treated with Tg (0.1 μM or 1 μM Tg) or DMSO for 60 hours (n=6 mice per sex, > 1000 cells per group). (A, B) Average change in fluorescence intensity from all GFP expressing female (A) and male (B) β cells over time. Data was normalized to the first 2 hours to examine relative change in *Ins2* gene activity. (C) Viability was assessed immediately following dispersions. There were no significant difference between males and females (p=0.1603; unpaired Student’s *t*-test).

**Figure S8. Mouse islet cell viability post dispersion.** Viability was assessed immediately following dispersions in 20-week-old male and female B6 mice. There were no significant differences between males and females (p=0.7707; unpaired Student’s *t*-test).

**Figure S9. Representative western blot images of UPR protein markers.** (A-D) Levels of ER stress proteins were quantified in isolated islets from 20-week-old male and female B6 mice cultured in DMSO or 1 μM Tg for 24 hours. (A) BiP levels were significantly upregulated in female Tg vs DMSO (p=0.0011; paired Student’s *t*-test) but not male Tg vs DMSO (p=0.1187; paired Student’s *t-*test). (B) pIRE1α levels were significantly upregulated in female Tg vs DMSO (p=0.0001; paired Student’s *t*-test) and in male Tg vs DMSO (p=0.0148; paired Student’s *t*-test). (C) CHOP levels were significantly upregulated in female Tg vs DMSO (p=0.0333; paired Student’s *t*-test) and in male Tg vs DMSO (p=0.0164; paired Student’s *t-*test). (D) p-eIF2α levels were not significantly upregulated in either sex. (E-G) Levels of ER stress proteins were quantified in isolated islets from 60-week-old male and female B6 mice cultured in DMSO or 1 μM Tg for 24 hours. (E) BiP levels were significantly upregulated in male Tg vs DMSO (p=0.0048; paired Student’s *t-*test) but not in female Tg vs DMSO (p=0.3319; paired Student’s *t*-test). (F) p-IRE1α levels were not significantly upregulated in either sex (p=0.9257 [female] and p=0.8273 [male]; paired Student’s *t-*test). (G) p-eIF2α levels were not significantly upregulated in either sex (p=0.8451 [female] and p=0.3076 [male]; paired Student’s *t-*test). (H) Representative western blot images of 20-week Tg treated mouse islets. (I) Representative western blot images of 60-week Tg treated mouse islets. * indicates p<0.05, ** indicates p<0.01; ns indicates not significant.

**Figure S10. Female mouse islets retain greater glucose-stimulated insulin secretion during ER stress.** (A) Insulin secretion at basal (3 mM; low glucose, LG) and stimulatory (20 mM; high glucose, HG) glucose. Female islet LG secretion was significantly higher compared with control after 2- and 4-hour Tg pre-treatments (p=0.0047 [2-hour] and p=0.0003 [4-hour]; Mann Whitney test). Female islet HG secretion was significantly higher compared with control after 0- and 2-hour Tg pre-treatments (p=0.0012 [0-hour] and p=0.0061 [2-hour]; Mann Whitney test). Male islet LG secretion was significantly higher compared with control after a 0-hour Tg pre-treatment (p=0.0371; Mann Whitney test). Male islet HG secretion was significantly lower compared with control after a 4-hour Tg pre-treatment (p=0.0012; Mann Whitney test). (B) Data from Figure S10A plotted to compare male vs female insulin secretion in high glucose. Male islet insulin secretion was significantly higher compared with females in control conditions (p=0.0012; Mann Whitney test) but not significantly different after Tg pre-treatments. (C) Data from Figure 4D plotted to compare male vs female insulin content. No significant differences between males and females under any treatment condition. (D) Proinsulin secretion at basal (3 mM) and stimulatory (20 mM) glucose. Female islet HG secretion was significantly higher compared with control after 0- and 2-hour Tg pre-treatments (p=0.0075 [0-hour] and p=0.0437 [2-hour]; Mann Whitney test). Male islet HG secretion was significantly lower compared with control after a 4-hour Tg pre-treatment (p=0.0025; Mann Whitney test). (E) Data from Figure S10D plotted to compare male vs female proinsulin secretion in high glucose. Male islet insulin secretion was significantly lower compared with females after 4-hour Tg pre-treatment (p=0.0106; Mann Whitney test). (F) Data from Figure 4E plotted to compare male vs female proinsulin content. No significant differences between males and females under any treatment condition. (G) Proinsulin/insulin content was not significantly different between males and females in any treatment condition * indicates p<0.05, ** indicates p<0.01; ns indicates not significant; error bars indicate SEM.

**Figure S11. Greater glucose-stimulated insulin secretion in** **Insr^f/f^:Ins1Cre^-/-^:nTnG^+/-^female mice.** (A-D) Physiology measurements after a 6-hour fast in 10 and 22-week-old male and female Insr^f/f^:Ins1Cre^-/-^:nTnG^+/-^ mice from (53). Insulin levels from glucose-stimulated insulin secretion tests (A, B: nM, C, D: % basal insulin) following a single glucose injection (2 g glucose/kg body weight, i.p). Area under the curve (AUC) calculations (10 weeks; n=17 females, n=15 males. 22 weeks; n=9 females, n=9 males). (A) No significant difference was identified between males and females insulin secretion values. (B) Insulin levels were significantly higher in male mice at all time points (p=0.004 [0 minutes], p=0.0188 [15 minutes] and p=0.0142 [30 minutes]; Mann Whitney test). AUC was significantly higher in males (p=0.0078; Mann Whitney test). (C) Insulin levels (% baseline). Glucose-stimulated insulin secretion was significantly higher in female mice 15- and 30-minutes post injection (p=0.0212 [15 minutes], p=0.0297 [30 minutes]; Mann Whitney test). AUC was significantly higher in females (p=0.0087; Mann Whitney test). (D) Insulin levels (% baseline). Glucose-stimulated insulin secretion was significantly higher in female mice 15- and 30-minutes post injection (p=0.0231 [15 minutes], p=0.0297 [30 minutes]; Mann Whitney test). AUC was significantly higher in females (p=0.0244; Mann Whitney test). (A-D) * indicates p<0.05, ** indicates p<0.01; ns indicates not significant; error bars indicate SEM. Because a Shapiro-Wilk test revealed the physiology data was not normally distributed, we used a Mann Whitney test. (E) Blood glucose measurements in mice fasted for 4 hours were monitored in insulin-reduced (InsKO) and control mice. n=5-39 per group. Male data was previously published (54); female data was collected in parallel but not published. * indicates timepoints at which we observed a sex:genotype interaction (see Supplementary file 7 for *p*-values).

**Figure S12. Mouse islet gene expression clusters by sex, treatment and time.** (A) Unsupervised hierarchical clustering of RNAseq data from female and male DMSO or Tg treated mouse islets. Sorting was based on all genes where the total count >10 across all samples.

**Figure S13. Female and Male mouse islets are enriched in similar pathways following 6- and 12-hour Tg treatments.** (A, B) Most significantly enriched Reactome pathways from the top 1000 significantly differentially expressed genes. (*p*-adj < 0.01) for females and males between DMSO vs Tg after 6 hours (A) or 12 hours (B) of Tg treatment. Gene ratio is calculated as k/n, where k is the number of genes identified in each Reactome pathway, and n is the number of genes from the submitted gene list participating in any Reactome pathway.

**Figure S14. Female and Male mouse islets share similar Tg-induced changes to mRNA levels of genes related to apoptosis following 6- and 12-hour Tg treatments.** (A, B) All transcripts of differentially expressed genes under the gene ontology terms “positive regulation of apoptotic process” (GO:0043065) and “negative regulation of apoptotic process ” (GO:0043066). Genes are labeled by their role in the positive or negative regulation of apoptosis. The fold change (Tg/DMSO) of male and female apoptotic related genes are shown following 6-hour Tg treatments (A) and 12-hour Tg treatments (B). Genes that are significantly different (*p*-adj < 0.01) between DMSO and Tg are indicated by green and yellow bars. Non-significant genes are shown in gray bars.

**Figure S15. A greater number of β cell identity genes are downregulated between 6- and 12- hour Tg treatment times in female mouse islets.** (A, B) Treatment:Time interaction plots of female islet (A) and male islet (B) β cell identity genes in Reactome pathway “Regulation of gene expression in β cells”. The fold change (FC) for DMSO vs Tg was calculated for each sex and time point (Female 6-hour, Female 12-hour, Male 6-hour, Male 12-hour). The change in FC values (12-hour FC – 6-hour FC) were plotted according to *p*-adj values. In females, FC values between 6- and 12-hours are represented by orange and purple dots, respectively. In males, FC values at 6- and 12-hours are represented by green and blue dots, respectively. A solid black line connecting the dots indicates genes with a significant treatment:time interaction.

**Figure S16. Analysis of the transcriptome and partial proteome.** Changes in gene expression and protein levels were compared 6-hours post Tg treatment. Log2 transformed fold change values were used to assess the congruence between our proteomics data and RNAseq data. (A) Females. (B) Males.
